# Biomechanical study of the fixation stability of broken pedicle screws and subsequent strategies

**DOI:** 10.1371/journal.pone.0219189

**Published:** 2019-06-28

**Authors:** Ming-Kai Hsieh, Mu-Yi Liu, Jin-Kai Chen, Tsung-Ting Tsai, Po-Liang Lai, Chi-Chien Niu, Ching-Lung Tai

**Affiliations:** 1 Institute of Biotechnology, National Taiwan University, Taipei, Taiwan; 2 Bone and Joint Research Center, Chang Gung Memorial Hospital, Taoyuan, Taiwan; 3 Department of Orthopaedic Surgery, Spine Section, Chang Gung Memorial Hospital and College of Medicine, Chang Gung University, Taoyuan, Taiwan; 4 Ph.D. Program in Biomedical Engineering, Collage of Engineering, Chang Gung University, Taoyuan, Taiwan; 5 Graduate Institute of Biomedical Engineering, Chang Gung University, Taoyuan, Taiwan; University Magna Graecia of Catanzaro, ITALY

## Abstract

Pedicles are often broken when screws are inserted into hard pedicles with small diameters or when the diameter of the screw itself is inadequate. However, there is a lack of biomechanical literature that addresses screw loosening as a result of broken pedicles or the resulting salvage of those screws. We performed a novel *in vitro* study to compare the pullout strength of screws between intact pedicles and two different types of broken pedicles; strategies to prevent screw loosening were also compared. Thirty L4 Sawbones were designed to represent intact pedicles, semi-pedicles, and nonpedicles and were prepared for screw insertion. Three sizes of polyaxial screws (diameter × length dimensions of 6.0 mm × 45 mm, 6.0 mm × 50 mm and 6.5 mm × 45 mm) were independently and randomly distributed into the intact-pedicle group (IP group, n = 30), the semi-pedicle group (SP group, n = 15), or the nonpedicle group (NP group, n = 15). The experiments were conducted across a minimum of five trials for each of the chosen screw sizes. We then analyzed the results of the imaging, pullout testing, and embedded bone volume. Any fractures or defects of the vertebrae caused by screw insertion were excluded from the study. The appropriate screw trajectory and insertional depth were confirmed using axial and sagittal X-ray imaging prior to screw pullout testing. A pullout strength of only 41% to 45% for the SP group and 29% to 39% for the NP group was retained following a broken pedicle. The use of longer or larger-diameter screws appears to be an effective salvaging procedure for the NP group (*p* < 0.05). The embedded bone volume percentage analysis indicated that, compared to the IP group, 68% to 76% of effective bone embedded into the screw threads in the SP group, and 58% to 65% in the NP group. There was no direct correlation between the pullout strength and the embedded bone volume; however, less effective embedded bone volume was associated with lower pullout strength. This study describes the evolution of the well-established screw pullout test being applied to the broken pedicle Sawbone testing model. The pedicle plays an important role in determining the pullout strength of a screw. However, a salvage procedure that utilizes a longer or larger-diameter screw might be a reliable clinical approach to address broken pedicles.

## Introduction

Pedicle screw fixation during spinal fusion surgery is a useful approach for treating spinal disorders, such as degenerative spinal diseases, spinal infection, or metastatic spinal lesions. The use of a pedicle screw as a spinal implant was first described by Boucher [1] in the 1950s and was reintroduced by Roy-Camille et al. in the 1980s [2] and represents a practical, safe, and superior approach to uninstrumented fusion in the treatment of various spinal conditions [3]. The incidence of screw loosening is between 0.6% and 11% [3–5] and may cause the fusions to become unstable, which can lead to serious postoperative complications and may require subsequent surgery. Screw loosening is a major concern, and radiolucency around screws is detrimental to fixation [6], often requiring revision surgery [7]. Broken pedicles result from the insertion of screws with inadequate diameters or inserting screws into hard pedicles with small diameters. Previous studies have focused on the salvage procedures for loosened screws in the osteoporotic spine that include the use of expandable screws, the extension of the instrumented level, the insertion of longer screws, exchanging screws for those with a larger diameter [8–11], improving the design of the screw-rod system [12–15], and the use of a cannulated pedicle screw with polymethylmethacrylate (PMMA) cement augmentation [16–23]. However, there is a lack of biomechanical literature addressing screw loosening as a result of broken pedicles or subsequent screw salvage. In this novel *in vitro* experimental study, we compared the pullout strength of screws between intact pedicles and two different kinds of broken pedicles, and compared subsequent strategies to prevent screw loosening.

## Materials and methods

### Specimen preparation and instrumentation

**Fig 1:**
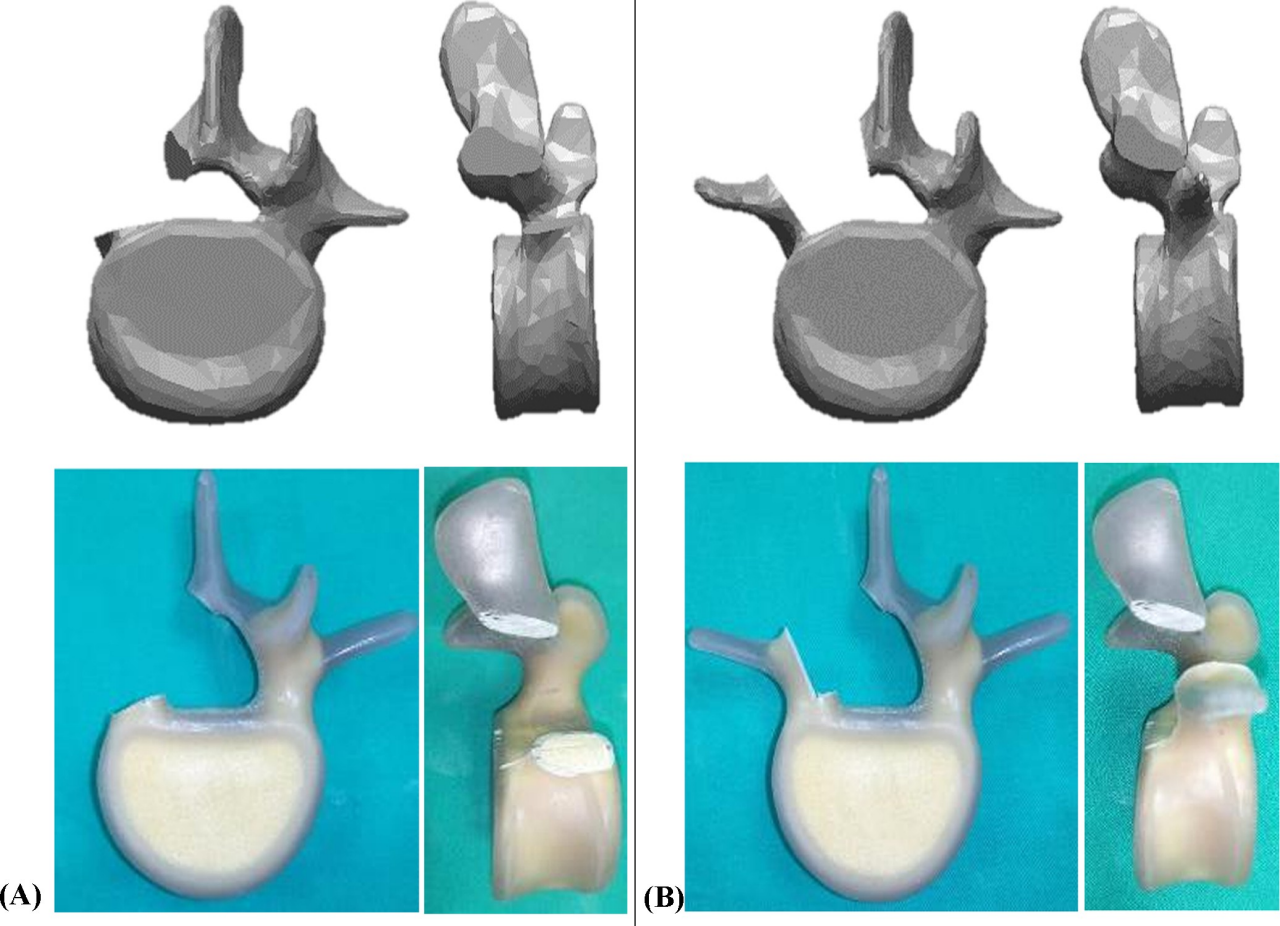
**Sawbones were designed to represent nonpedicle (A, left side), semi-pedicle (B, left side), and intact pedicle (A and B, right side).** The designed computer simulated Sawbones models were shown in the upper row; the real designed Sawbones models were in the lower row.

**Fig 2:**
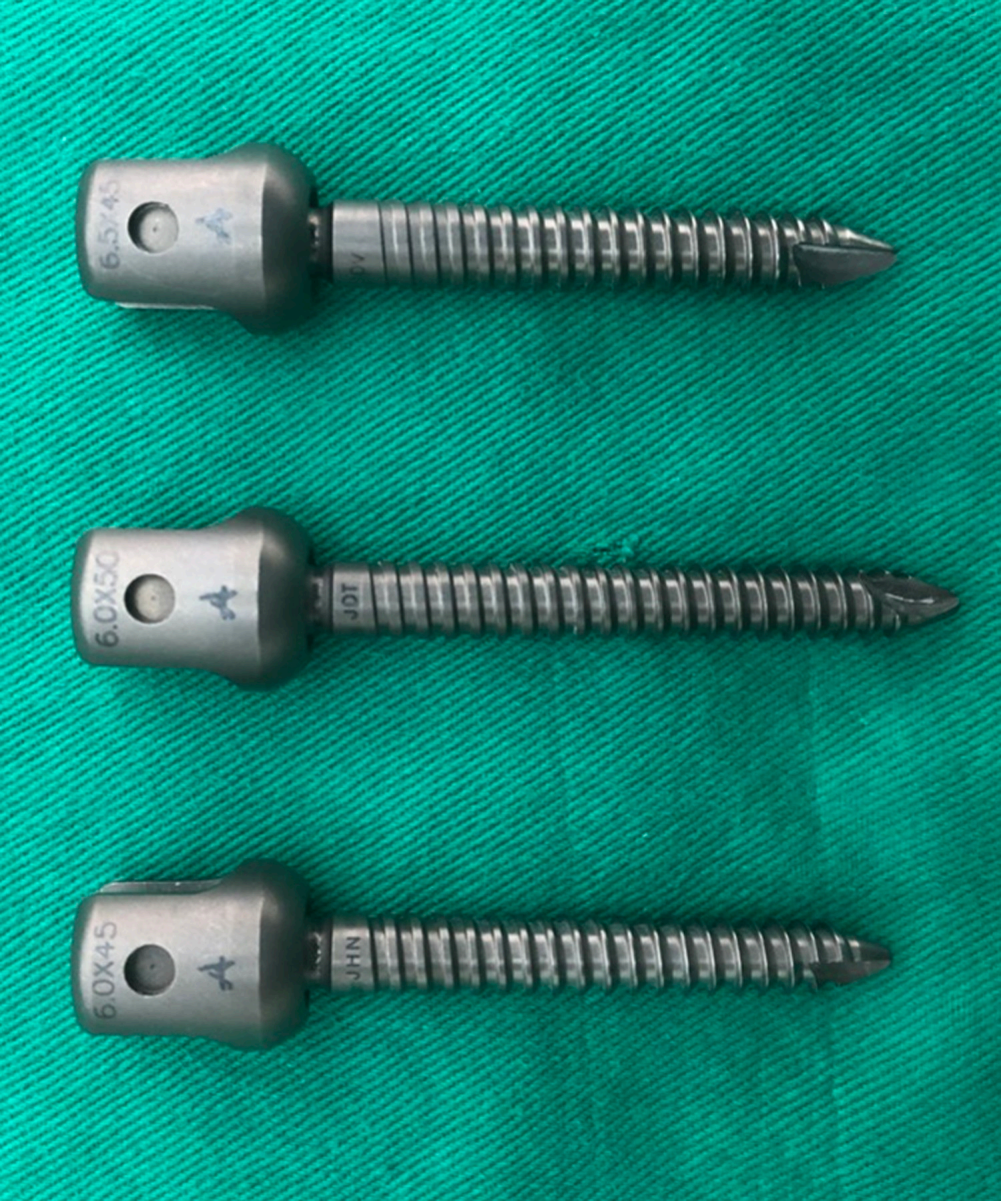
Photographs showing the three types of poly-axial screws used in the study. **Left to right: 6.0 mm × 45 mm, 6.0 mm × 50 mm, and 6.5 mm × 45 mm (diameter × length).** The length was measured from the point of the bottom of the hub to the screw tip. The diameter was defined as the outer diameter. All screw types had a thread pitch of 1 mm and a thread depth of 0.8 mm.

**Fig 3:**
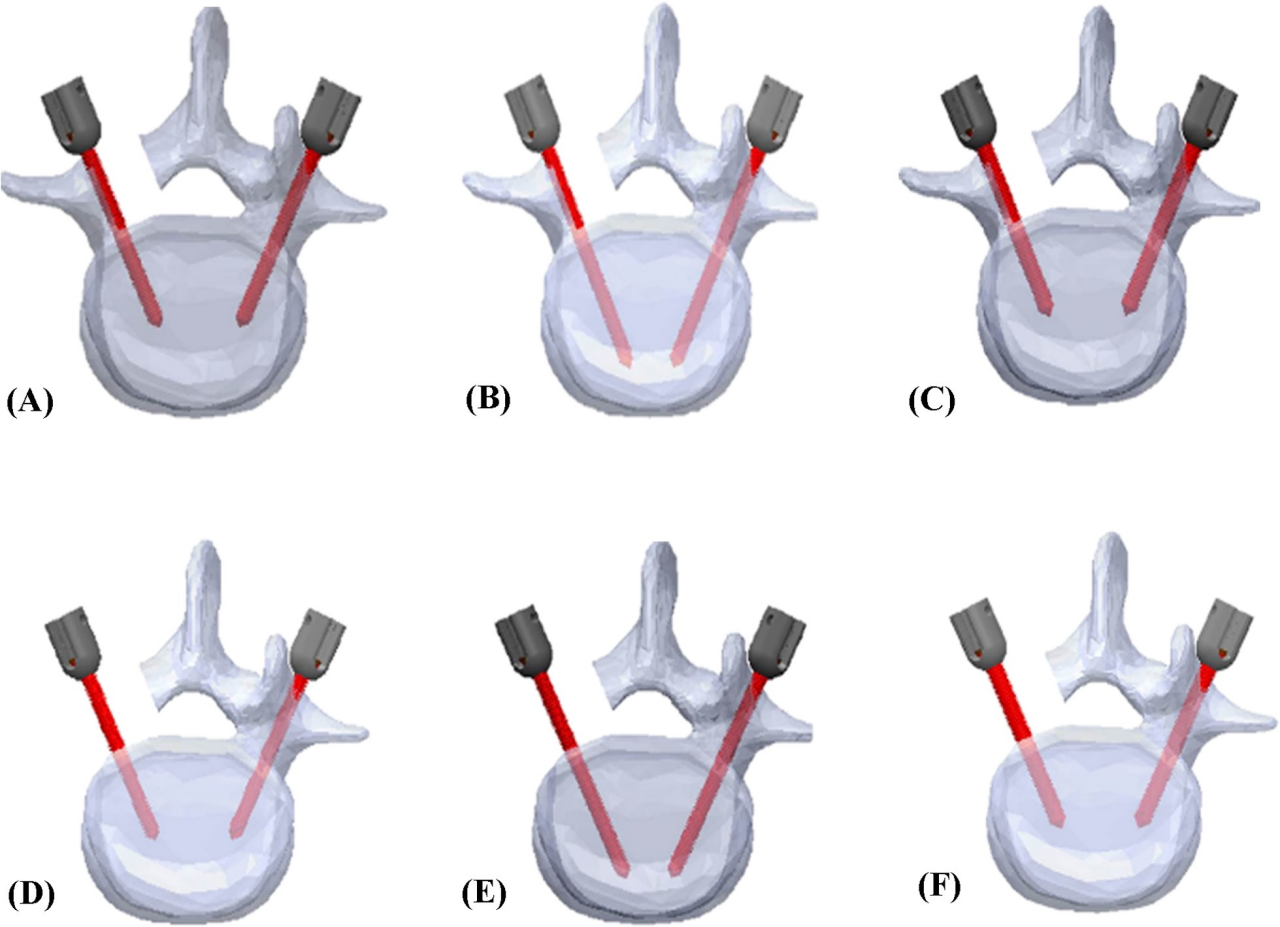
Schematic drawing showing the designation of synthetic vertebrae instrumented with various pedicle screws. Different-sized screws were chosen and randomly implanted into each pedicle of the vertebrae by an experienced surgeon, 6.0 mm × 45 mm, 6.0 mm × 50 mm, and 6.5 mm × 45 mm screws were inserted sequentially into the semi-pedicle (left side of A, B, C) and nonpedicle (left side of D, E, F). The screws were implanted into the right side pedicle as the intact pedicle group (A-F).

Thirty L4 Sawbones (Model: #3429–4, Pacific Research Laboratory Inc., Vashon Island, Washington, USA) that were specifically designed to be radio-opaque under fluoroscopy were prepared for screw insertion. The Sawbones have an inner cancellous core and an outer cortex layer, which simulate human spine vertebrae with normal bone properties and morphometry. The specimens were designed to represent either an intact pedicle, a semi-pedicle, or a nonpedicle, illustrated in Fig 1. A pilot hole was drilled using a 2.5 mm “twist” metric drill bit attached to a Dremel 4000 rotary tool that was mounted on a Dremel WorkStation Model 220–01. Each vertebra was placed in a small clamp and oriented so that the drill tract was horizontal to the drill pin. This trajectory was selected based on previously reported morphometric characteristic data [24]. The pilot track was followed with a standard straight pedicle probe to a depth of 45 mm. Three sizes of polyaxial screws (diameter × length dimension of 6.0 mm × 45 mm, 6.0 mm × 50 mm, and 6.5 mm × 45 mm) (SmartLoc spinal polyaxial pedicle screws, A-spine Asia Co. Ltd., Taipei, Taiwan) (Fig 2) were chosen and randomly implanted into each pedicle of the vertebrae by an experienced surgeon. Then, the specimens were randomly distributed into the intact-pedicle group (IP group, n = 30), the semi-pedicle group (SP group, n = 15), or the nonpedicle group (NP group, n = 15). These experiments were conducted in five trials for each screw size (Fig 3). The 6.0-mm diameter and 45-mm length screws were chosen based on feedback from experienced surgeons who indicated that this is the most commonly utilized size in the clinic.

### Image analysis

Axial and sagittal views were examined via X-ray for all specimens prior to the pullout test to confirm an appropriate screw trajectory and insertion depth. The vertebrae were also examined thoroughly to rule out any fractures or defects caused by screw insertion.

### Pullout test

**Fig 4:**
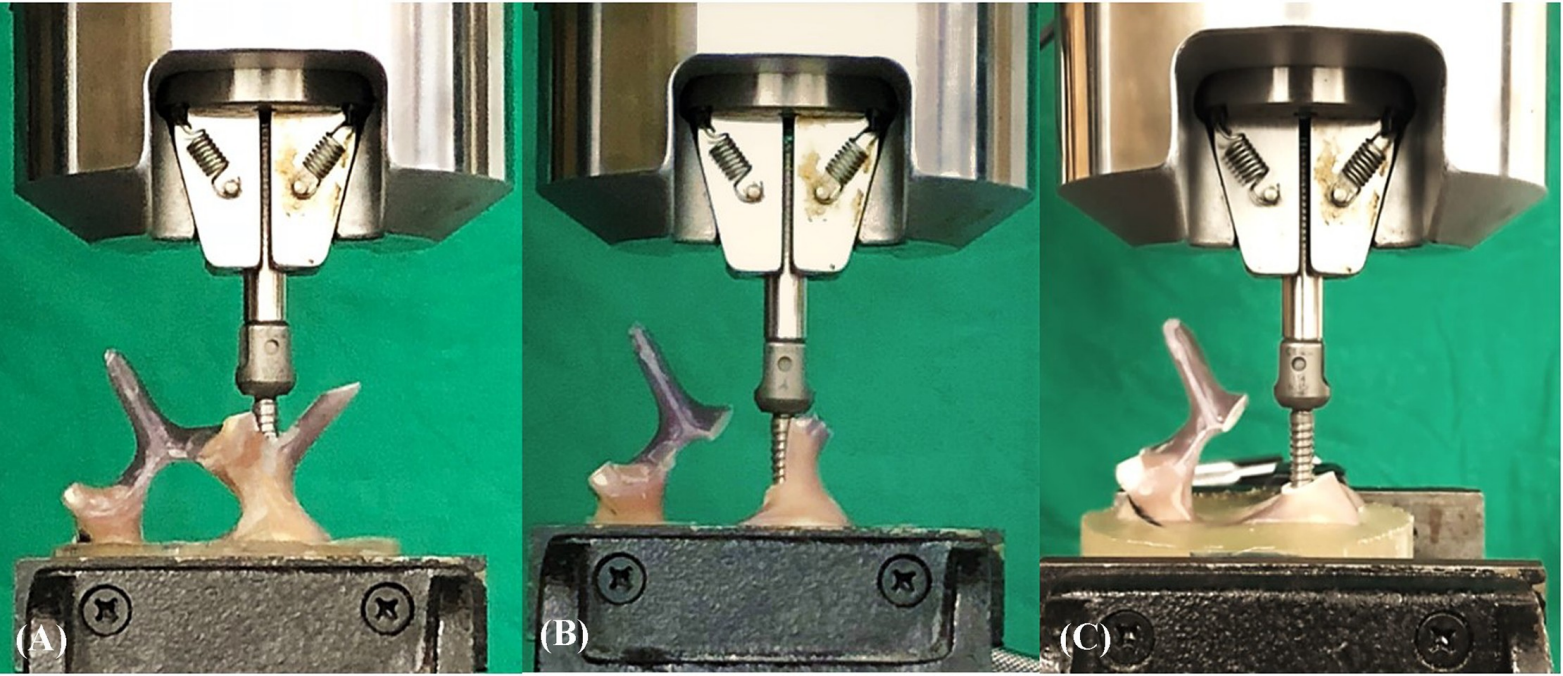
Photograph showing the experimental setup of the screw pullout test. Sawbones were potted in specific epoxy resins. The prepared specimens were mounted onto a material testing machine (Bionix 858; MTS Systems Corp., MN, USA) to conduct the axial pullout tests with the (A) intact pedicle screw, (B) semi-pedicle screw, and (C) nonpedicle screw.

Each of the 30 Sawbones was potted in metal boxes using specific epoxy resins (Buehler, Lake Bluff, IL, USA). Judicious potting was performed to ensure that the cement did not come into contact with any portion of the pedicle screw. The prepared specimens were mounted onto a material testing machine (Bionix 858; MTS Systems Corp., MN, USA) to conduct axial pullout tests with the screws (Fig 4). The polyaxial screw head was fixed to a 10-mm diameter cylindrical rod with an outer thread that matched the inner thread of the screw head. The cylindrical rod was then clamped to the upper wedge grip of the MTS testing machine. The potted specimen was secured on a lower custom-made grip capable of x-y plane translation and rotation to achieve the coaxial alignment of the pedicle screw with the pullout ram. We ensured that the pedicle screws and the pullout force were directed along the same axis by clamping the potted specimen in a way that enabled free translation in the x-y plane and free rotation about the x-axis, and the direction of the pullout force could be adjusted through the polyaxial design of the screw head. The screws were then loaded in displacement control mode at a constant displacement rate of 5 mm/min for a total displacement of 10 mm, which is in accordance with published literature on axial pull out testing [14,25,26]. Data collection was set at 1 sample/0.05 mm (1.67 Hz). Failure was defined as the maximum load or the load peak prior to a decrease in load associated with increasing displacement [25–27]. After the pullout test was completed, the specimen and the screws were closely examined for signs of fracture and damage and any findings were carefully recorded.

### Embedded bone volume (EBV)

**Fig 5:**
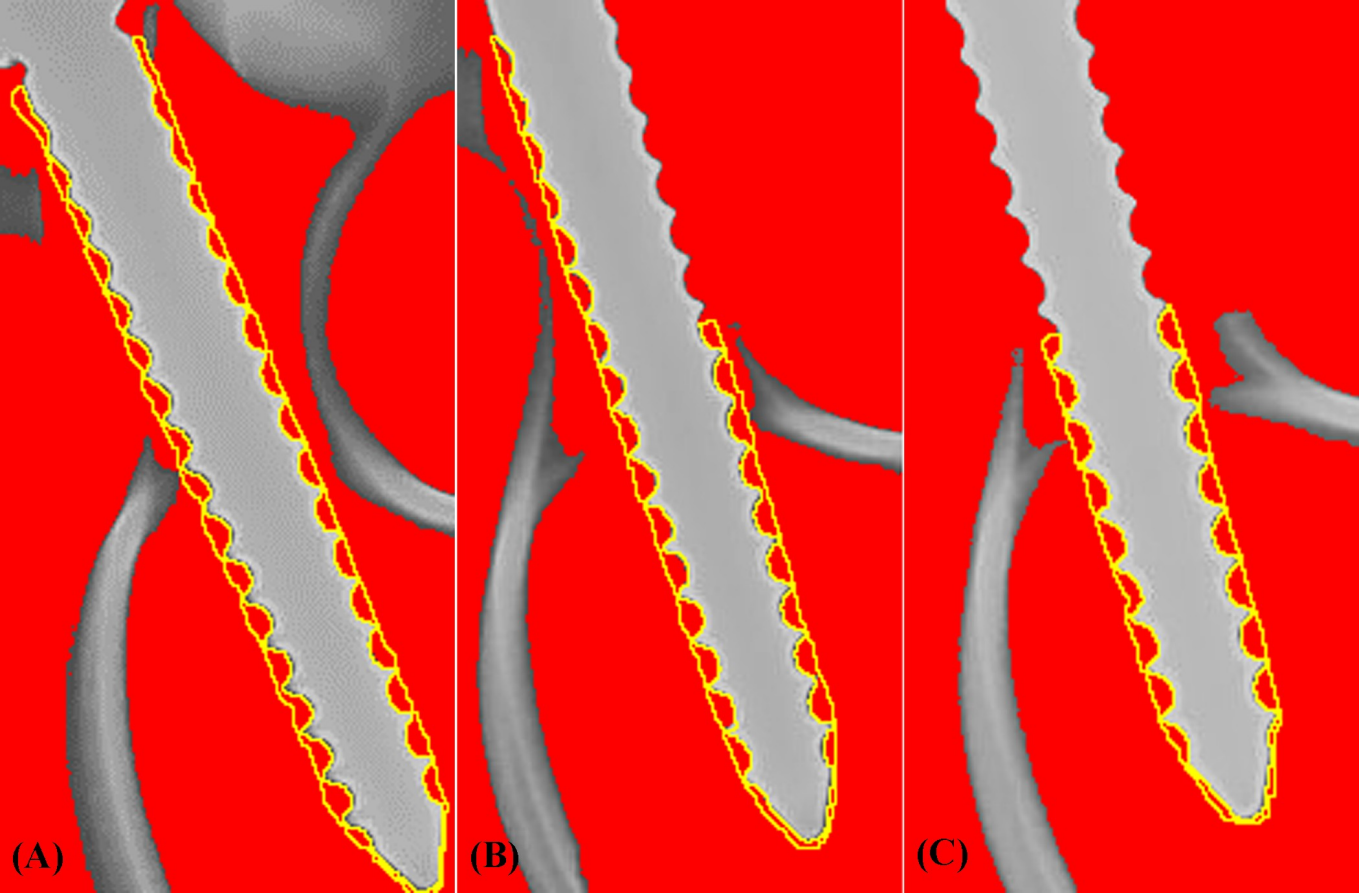
Photographs showing the highlighted area of bone that was embedded into the screw thread using radiographic imaging of inserted screws and ImageJ analysis software. (A) Intact pedicle group, (B) semi-pedicle group, and (C) nonpedicle group. The bone volume embedded into the screw thread was quantified and defined as $EBV=A\times \frac{\pi (D+d)}{2}$, where “A” denotes the calculated area of bone embedded into the screw thread from the 2-D radiographic image, and “D” and “d” denote the outer and inner diameter of the pedicle screw, respectively.

The bone volume embedded into the screw thread was quantified by analyzing the radiographic image of the inserted screw with image analysis software (ImageJ, National Institutes of Health, Bethesda, Maryland, USA), and is defined as follows:

$$EBV=A\times \frac{\pi (D+d)}{2}$$

where “A” denotes the calculated area of bone embedded into the screw thread from a 2-D radiographic image, and “D” and “d” denote the outer and inner diameter of the pedicle screw, respectively (Fig 5). To investigate the association between pullout strength and embedded bone volume, the percentage of embedded bone volumes for the SP and NP groups were compared, where the percentage of embedded bone volume (SP or NP) was defined as the embedded bone volume (SP or NP) divided by the embedded bone volume for the IP group.

### Statistical analysis

The measurements for the semi-pedicle and nonpedicle groups of each screw size were collected in five trials. Moreover, measurements for the intact-pedicle groups of each screw size were collected in ten trials, and expressed as the mean ± standard deviation (SD). Statistical software (SPSS for Windows version 12.0, SPSS Inc., Chicago, IL) was used to analyze the pull out strength and embedded bone volume of all specimens. All of the measurements were collected for 30 vertebrae and expressed as the mean ± standard deviation (SD). An ANOVA test with post hoc analyses was performed to evaluate the differences between groups. Differences were considered to be significant at *p* < 0.05.

## Results

### Image analysis

**Fig 6:**
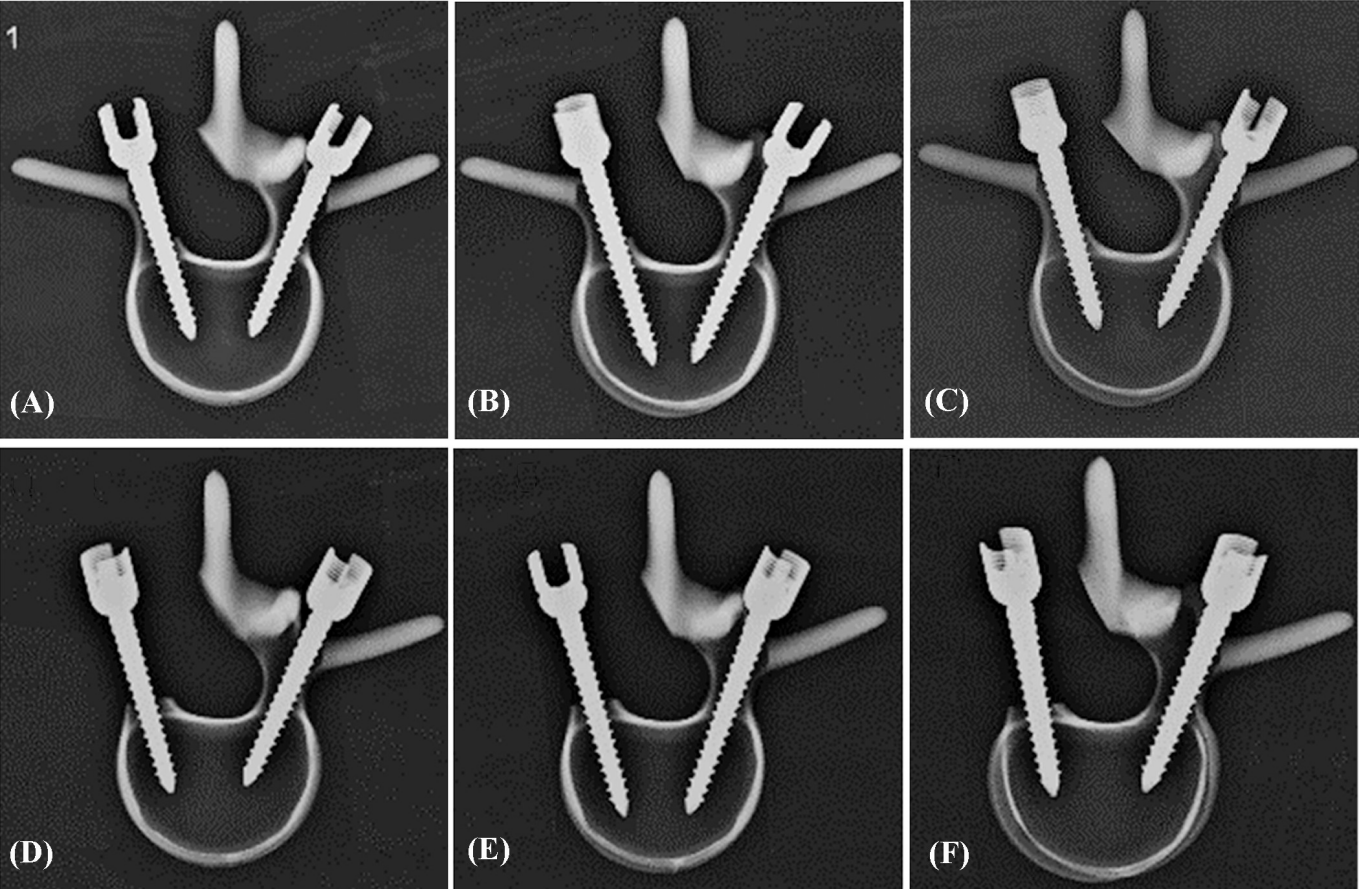
**Axial X-ray images show that the 6.0 mm × 45 mm, 6.0 mm × 50 mm, and 6.5 mm × 45 mm screws were inserted sequentially into the semi-pedicle (left side of A, B, C), nonpedicle (left side of D, E, F), and intact pedicle (right side of A-F).** An appropriate screw trajectory and insertional depth were confirmed using axial X-ray imaging prior to the pullout test. In the axial view, all screws were convergently inserted into the vertebral body, and the additional 5 mm of depth was clearly noted in the 6.0 mm×50 mm group (B,E) when compared to the other two groups (A, C, D, F).

**Fig 7:**
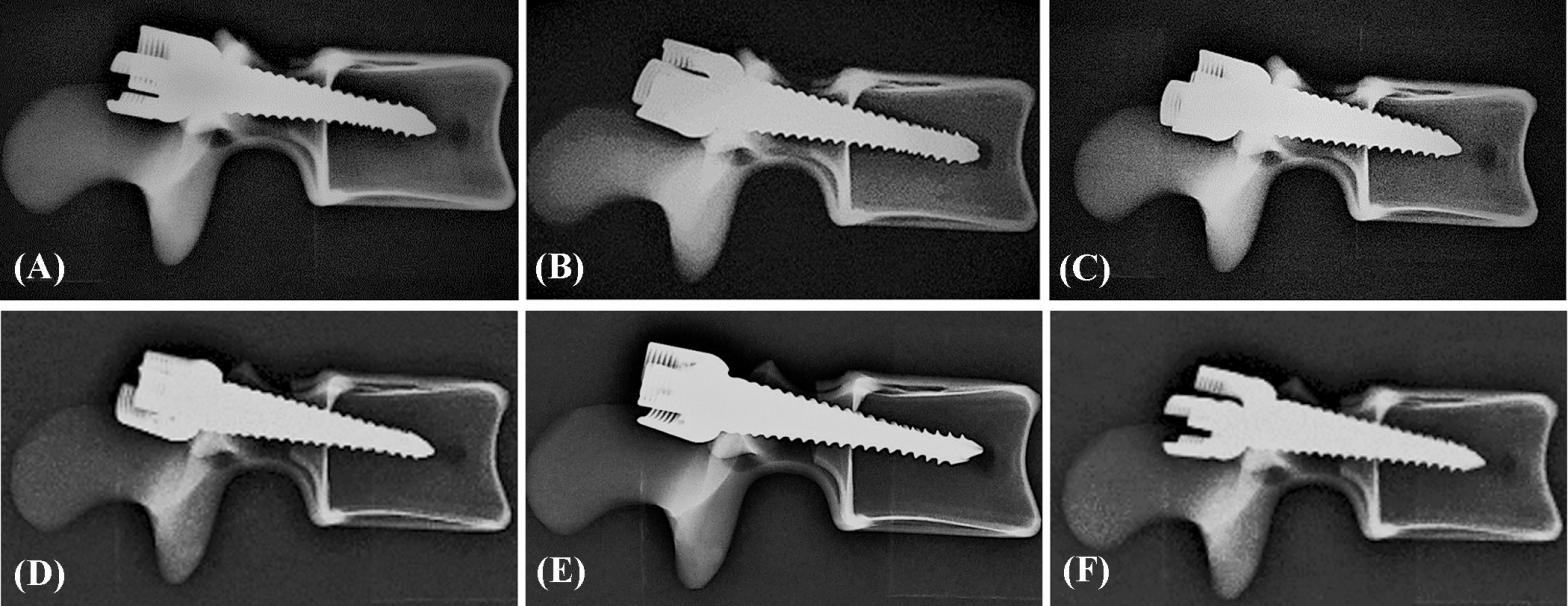
**Sagittal X-ray images show that the 6.0 mm × 45 mm, 6.0 mm × 50 mm, and 6.5 mm × 45 mm screws were inserted sequentially into the semi-pedicle (left side of A, B, C), nonpedicle (left side of D, E, F), and intact pedicle (right side of A-F).** All screws were inserted in a slightly downward orientation and both screws in the same specimen were parallel, consistent with current surgical techniques. No fractures or defects were detected across all vertebrae.

An appropriate screw trajectory and insertional depth was confirmed using axial and sagittal X-ray imaging prior to pullout testing (Figs 6 and 7). In the axial view, all screws were convergently inserted into the vertebral body, and the additional 5 mm of depth was observed clearly in the 6.0 mm × 50 mm group when compared to the other two groups (Fig 6). In the sagittal view, all screws were inserted in a slightly downward orientation and both screws in the same specimen were parallel, which is consistent with current surgical techniques (Fig 7). No fractures or defects in the vertebrae were detected in either view.

### Pullout strength

**Fig 8:**
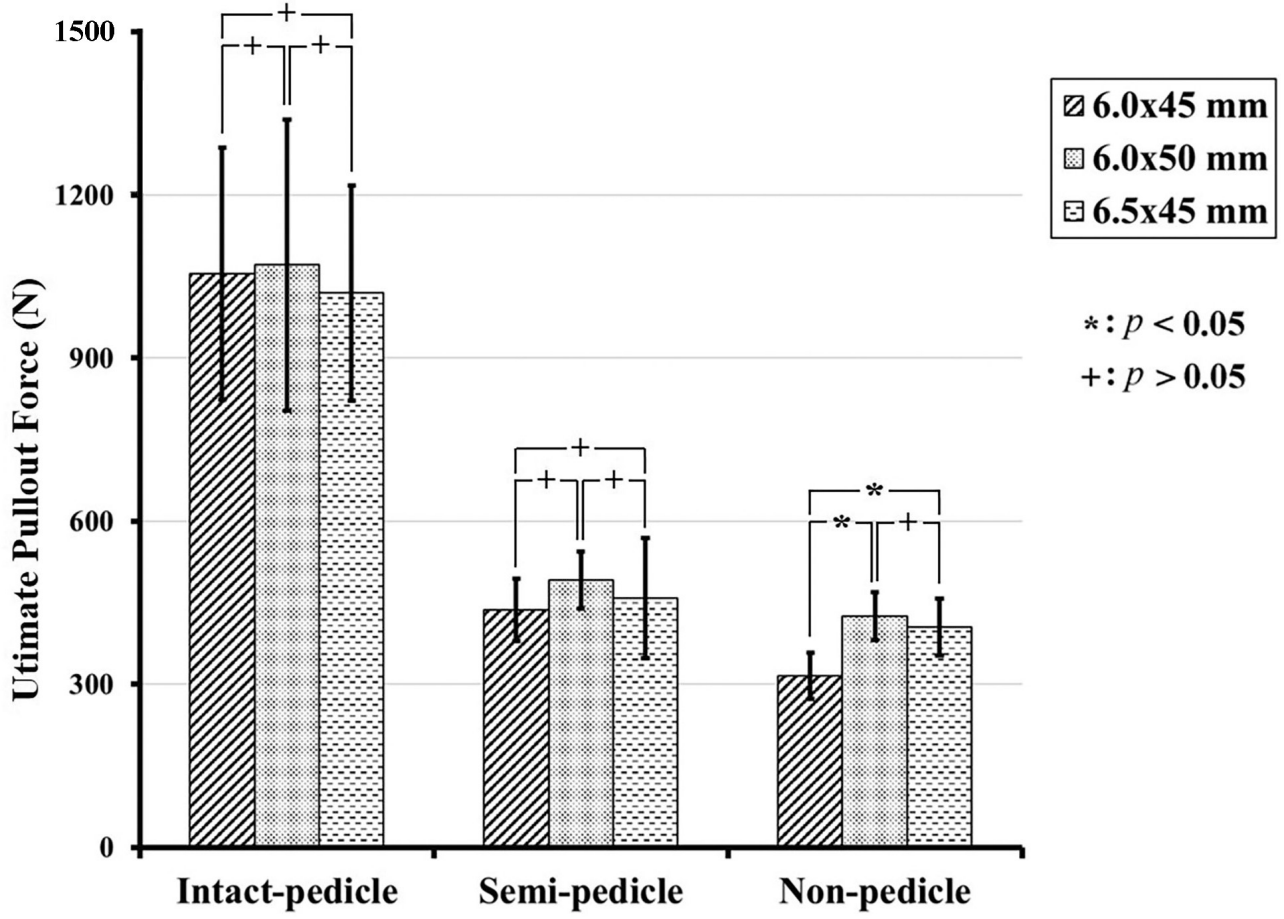
Mean ultimate pullout forces for various sizes of screws in each group. The vertical lines represent the standard deviations. Pullout forces of various sizes were not significantly different from each other in the intact and semi-pedicle groups. In contrast, the pullout force of the longer and larger screws were significantly higher in the nonpedicle group (*p* < 0.05).

**Fig 9:**
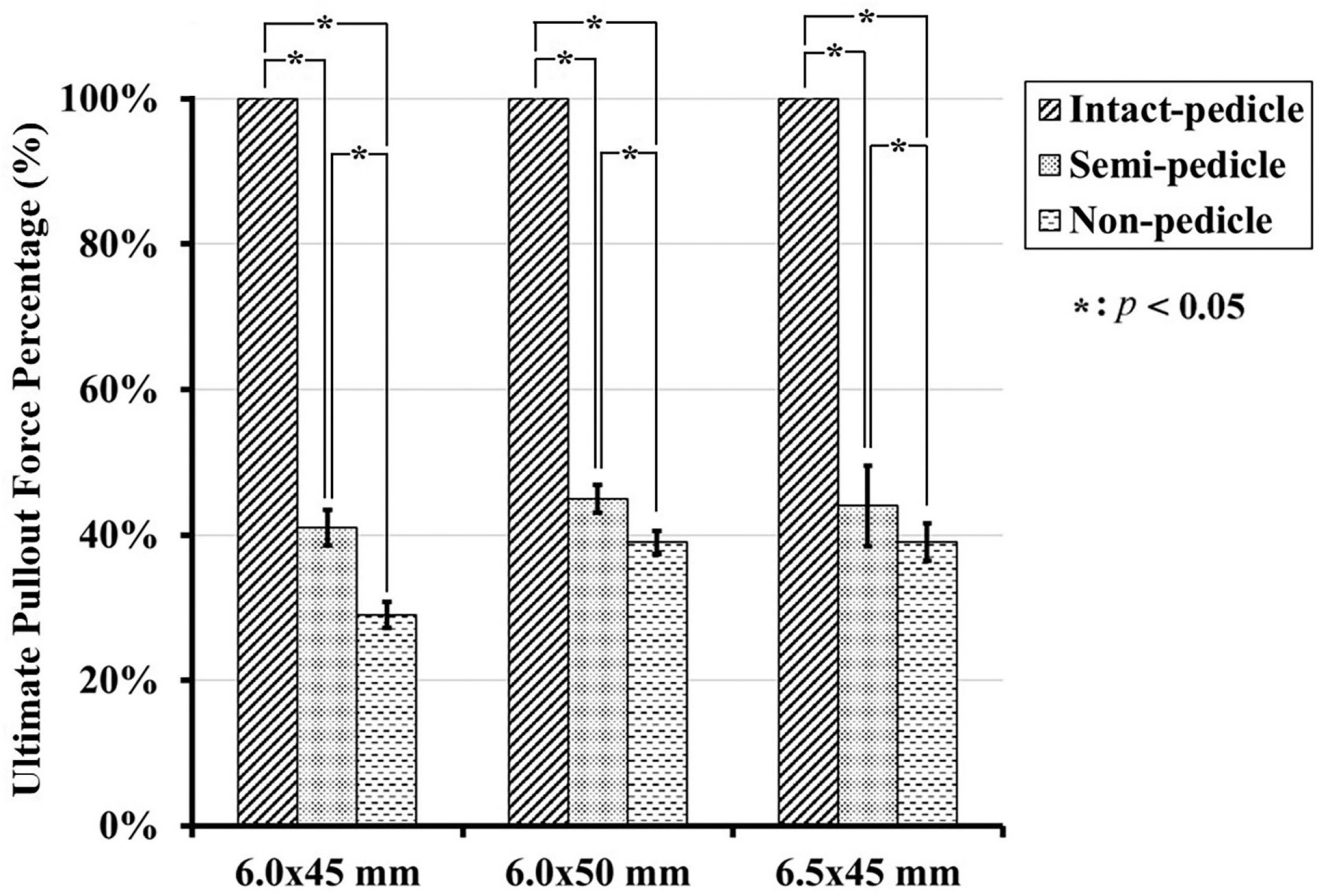
Maximum pullout force percentage for each screw size in the three groups. Significantly higher pullout strengths were demonstrated for all three screw sizes in the intact pedicle group when compared to the semi-pedicle or nonpedicle groups (*p* < 0.05). Higher pullout strengths were also measured in the semi-pedicle group when compared to the nonpedicle group for all sizes (*p* < 0.05).

**Fig 10:**
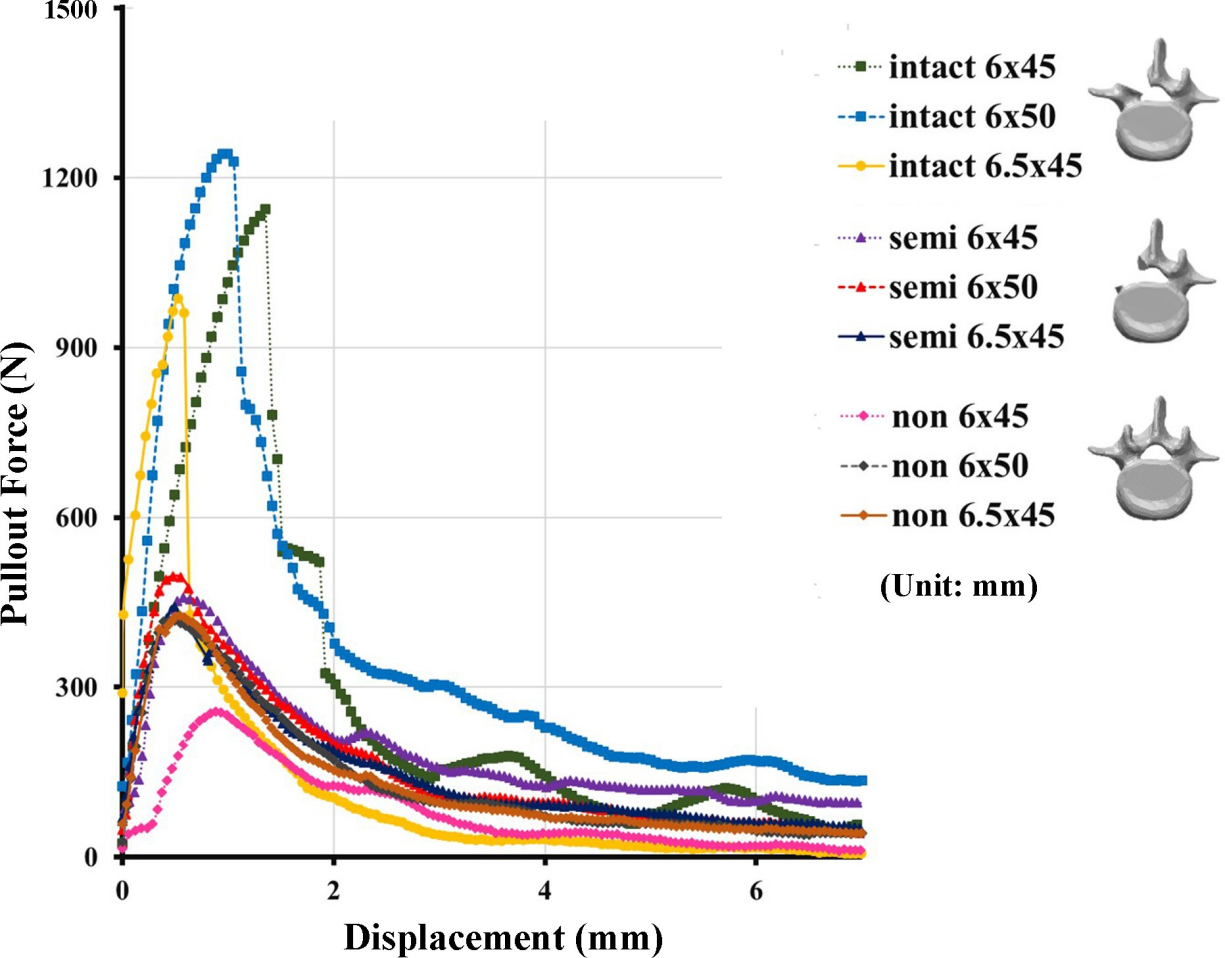
Typical force-displacement curves for the intact pedicle, semi-pedicle, and nonpedicle groups when instrumented with various sizes of pedicle screws.

The 6.0 mm × 45 mm, 6.0 mm × 50 mm, and 6.5 mm × 45 mm screws had mean pullout strength values of 1054.88 ± 231.9 N, 1071.25 ± 267.19 N, and 1019.52 ± 198.68 N, respectively, in the IP group; 436.2 ± 57.7 N, 469.69 ± 52.76 N, and 457.98 ± 110.61 N, respectively, in the SP group; and 314.89 ± 42.82 N, 424.76 ± 43.90 N, and 405.63 ± 52.23 N, respectively, in the NP group (Figs 8–10).

The pullout strengths of the various sized screws were not significantly different from each other in the intact pedicle and semi-pedicle groups. In contrast, the pullout strengths of the longer and larger-diameter screws were significantly higher in the nonpedicle group (*p* < 0.05) (Fig 8). Significantly higher pullout strengths were demonstrated in the intact pedicle groups than in the semi-pedicle and nonpedicle groups for all three screw sizes (*p* < 0.05). Higher pullout strengths were also measured in the semi-pedicle groups than in the nonpedicle groups for all sizes (*p* < 0.05). The mean maximum pullout force of the specimens treated with longer (6.0 mm × 50 mm) or larger-diameter (6.5 mm × 45 mm) screws presented as percentages of the standard (6.0 mm × 45 mm) screw is illustrated in Fig 9. Pullout strengths of only 41% to 45% in the SP group and 29% to 39% in the NP group for all three screw sizes were observed. The typical force-displacement curves for the IP, SP and NP groups treated with various screws are shown in Fig 10. Regardless of the screw dimensions (6.0 mm × 45 mm, 6.0 mm × 50 mm, or 6.5 mm × 45 mm), the intact-pedicle group exhibited significantly higher pullout strength than the broken pedicle groups (semi-pedicle or nonpedicle).

### Embedded bone volume

**Fig 11:**
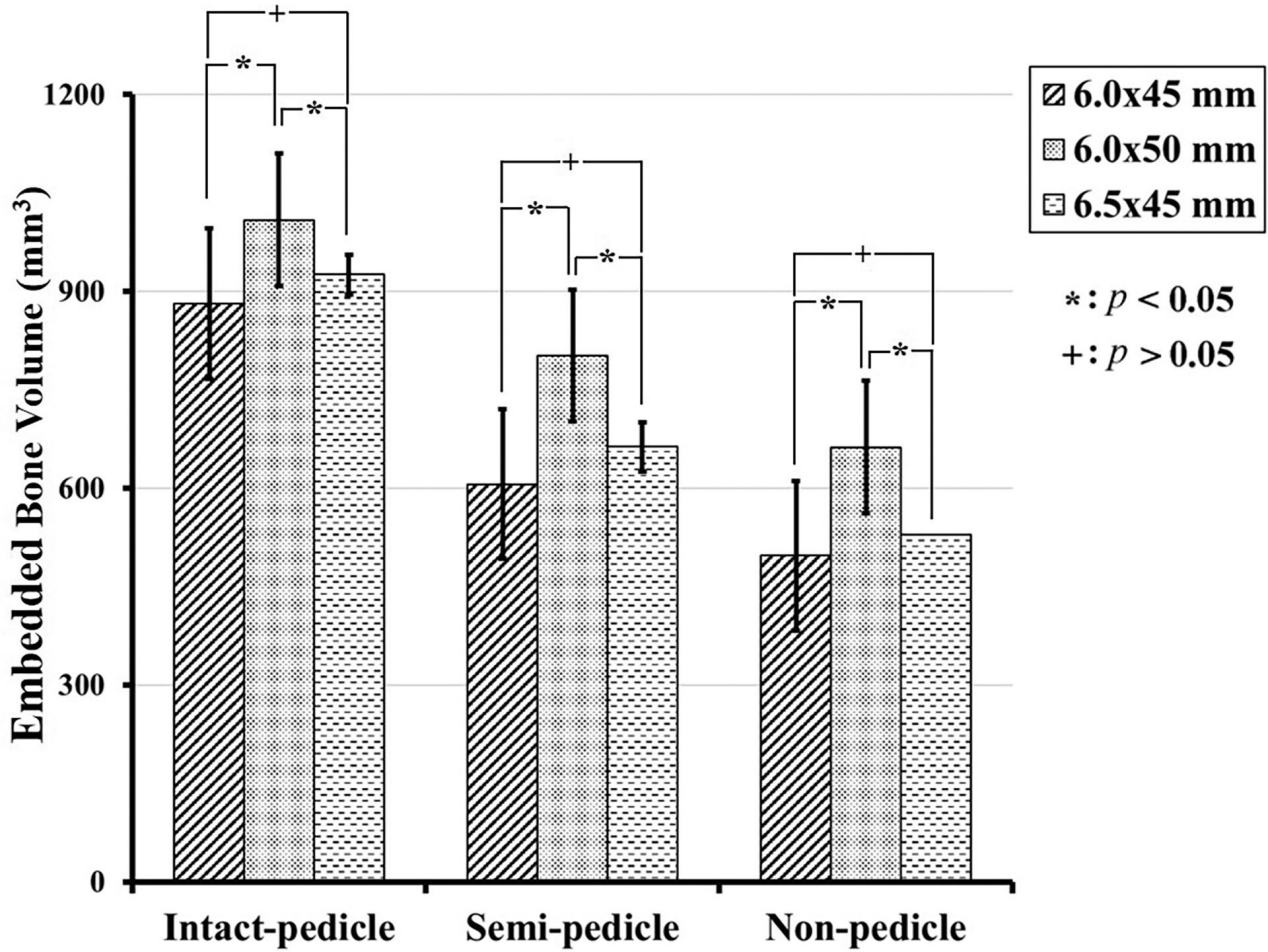
Embedded bone volumes quantified by ImageJ software using axial X-ray. In the IP group, the 6.0 mm × 45 mm, 6.0 mm × 50 mm, and 6.5 mm × 45 mm screws had a mean embedded bone volume of 880.88 ± 42.51 mm^3^, 1008.62 ± 46.6 mm^3^, and 925.34 ± 50.04 mm^3^, respectively, compared to 605.71 ± 30.53 mm^3^, 801.88 ± 40.62 mm^3^, and 662.59 ± 40.51 mm^3^, respectively, in the SP group, and 496.71 ± 22.94 mm^3^, 662.06 ± 30.58 mm^3^, and 529.12 ± 38.01 mm^3^ in the NP group.

**Fig 12:**
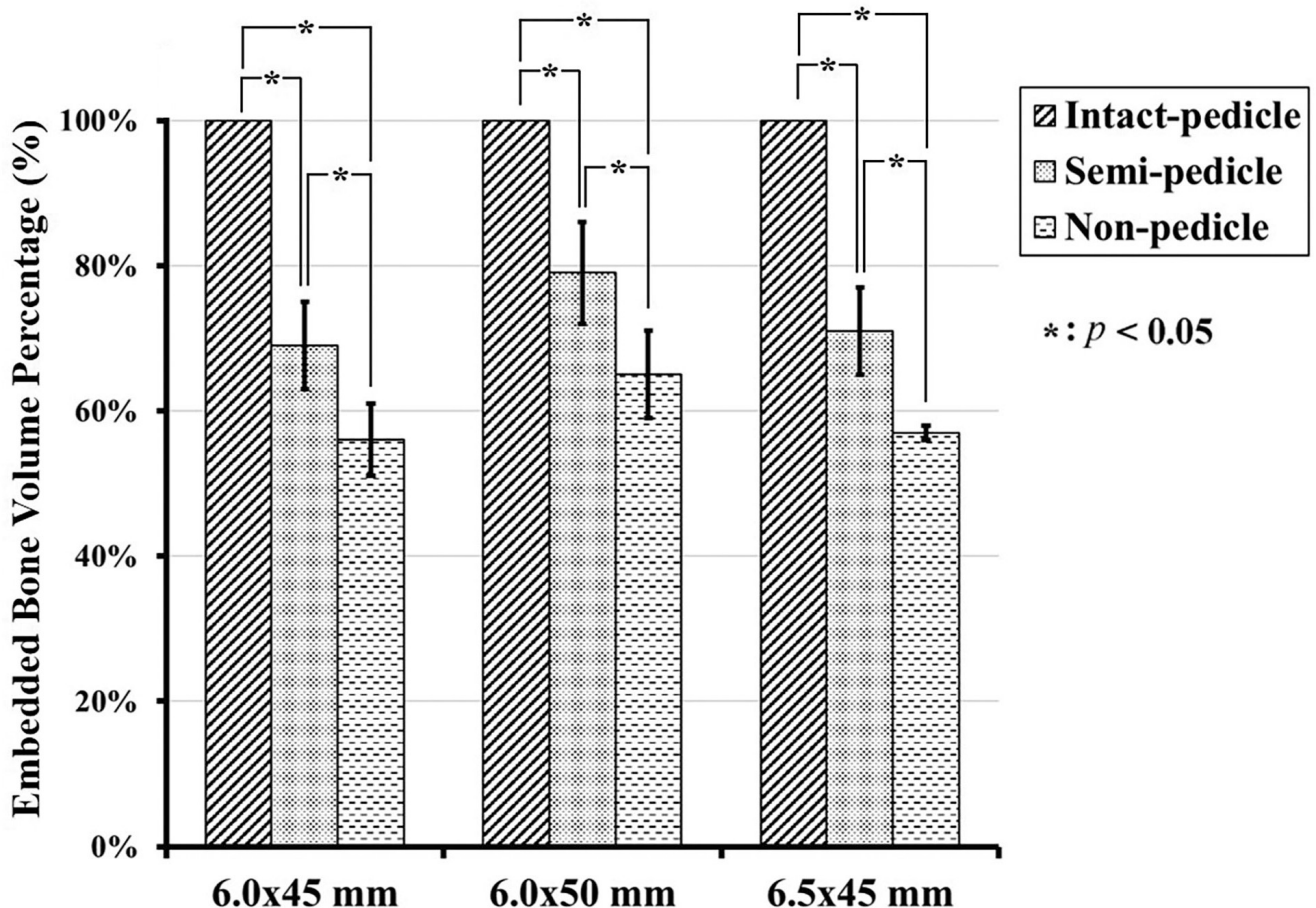
Embedded bone volume percentages of the three groups for each screw size. The value is significantly higher for the IP group than for the other groups and there is no statistically significant difference between the SP group (68–76%) and the NP group (58–65%). The distribution of these values is quite similar to those of the ultimate pullout forces (Fig 9).

The 6.0 × 45 mm, 6.0 × 50 mm, and 6.5 × 45 mm screws had mean embedded bone volumes of 880.88 ± 42.51 mm^3^, 1008.62 ± 46.6 mm^3^, and 925.34 ± 50.04 mm^3^, respectively, in the IP group; 605.71 ± 30.53 mm^3^, 801.88 ± 40.62 mm^3^, and 662.59 ± 40.51 mm^3^, respectively, in the SP group; and 496.71 ± 22.94 mm^3^, 662.06 ± 30.58 mm^3^ and 529.12 ± 38.01 mm^3^, respectively, in NP group (Fig 11). The embedded bone volume for the 6.0 × 50 mm screws is significantly higher than that of the 6.5 × 45 mm and 6.0 × 45 mm screws for all three groups, which is partially compatible with the pullout strength shown in Fig 8. The percent of embedded bone volume indicates the effective amount of bone that embedded in screw thread following a broken pedicle, which was recorded at 68–76% in the SP group and 58–65% in the NP group (these values are reported as percentages of the IP group results) (Fig 12). The amount of embedded bone is significantly higher in the IP group than in the other two groups, and there is no statistically significant difference between the SP and NP groups, which is compatible with the pullout strength shown in Fig 9.

## Discussion

Pedicle screw fixations are the gold standard for fixation surgeries and are widely used to treat spine instability and for other corrective surgeries. Pedicle breaks occur in normal-density bone when screws with inadequate diameters are inserted or when screws are inserted into hard pedicles with small- diameters. It is important to know the percentage-loss in pullout strength when a pedicle is broken and after salvage procedures are performed.

Synthetic bone models, such as Sawbones and polyurethane foam, are widely accepted due to their homogeneity and reproducibility compared to cadaveric samples and are well-established bone surrogates for biomechanical testing [28,29]. The Sawbones model provides physical strength properties that are more similar to those of the real spine than polyurethane foam, especially in studies in which anatomical simulation factors are important.

Numerous *in vitro* experiments have been conducted to improve screw fixation strength using polyurethane test blocks, and have suggested that these synthetic bones provide a useful platform for mechanical comparison of various designs of orthopedic devices [30,31]. However, the test blocks are rectangular in shape without consideration of actual bone morphology, which may have an impact on the reliability of the results in these studies. To the best of the authors’ knowledge, no existing research has addressed the effect of the absence of pedicles on screw anchoring power using synthetic bones with standard dimensions rather than test blocks. The present study is the first to develop and test a standard model that specifies conditions in the absence of pedicles on a compatible comparison platform. Our work is an *in vitro* experiment of specimens prepared in a laboratory environment, which does not necessarily represent clinical circumstances. In the present study, the assessment of screw fixation stability is limited to transverse axial screw pullout without consideration of other physiological loadings. However, in actual physiological situations, the pedicle screw is exposed to complex dynamic multidirectional loading. Although our results regarding the screw pullout mode did not represent actual physiological loading conditions, pullout tests are extensively used to assess the maximal forces that a pedicle screw can withstand and are considered an effective method to compare the relative screw fixation stability following screw insertion [21–23,25,27].

Previous studies addressing the association between vertebral morphology and screw fixation strength can be compared to this study. Weinstein et al. [32] summarized that approximately 60% of the pullout strength and load-to-failure of the thoracic and lumbar pedicle are in the pedicle itself, while the cancellous bone in the vertebral body adds another 15–20% strength and purchase in the anterior cortex offers another 20–25% increase. However, these researchers did not confirm this quantitatively with biomechanical experiments. Hirano et al. [33] performed a biomechanical test using forty-three lumbar vertebrae from the embalmed cadavers of 12 subjects. Groups were divided into screws that were placed in both the pedicle and vertebral body and screws that were placed only in the pedicle. They found that approximately 80% of caudocephalad stiffness and 60% of the pullout strength of the pedicle screw depended on the pedicle itself rather than the vertebral body. However, there were two major drawbacks of their study. First, wide variations in bone mineral density and the pedicle anatomy of cadaveric vertebrae might have an impact on the reliability of the results of the biomechanical investigation, even with randomized grouping. In the present study, standard L4 Sawbones were used as substitutes for cadaveric vertebrae, which eliminates the effects of the variability in bone properties and morphometry. Second, they reported that the pullout strength of the pedicle screw in the pedicle-only group was 60% of that for the pedicle-and-body group. However, their experimental designation for vertebrae with pedicle-only but without vertebral bodies does not exist in actual clinical applications. Pelletier MH et al. [34] used a “hubbing” technique that involved the removal of the cortical bone around the inserted screw and aimed to improve fixation and decrease screw loosening. These researchers concluded that the cortex plays a considerable role in protecting the underlying cancellous bone and contributing to the initial pullout strength. In their study, the “hubbing” technique itself is highly variable, and the removal of cortical bone around the screw could not predict the role of the pedicle, whereas in our study, we used the semi-pedicle and nonpedicle to standardize the pedicle defect.

Prior to this study, there was a lack of biomechanical reports addressing the pullout strength of pedicle screws in both intact and broken pedicles and the subsequent salvage strategies utilized to prevent implant failure due to broken pedicles. In our study, we found that the average pullout strength after complete (NP group) or partial (SP group) pedicle removal was only 29%-39% and 41%-45% of the intact pedicle, respectively (Fig 9), and even the use of larger-diameter or longer screws achieved values less than 50%. As shown in Fig 8, the pullout strengths of longer and larger-diameter screws were significantly higher in the nonpedicle group, but the same phenomenon was not observed in the other two groups. This finding indicates that the use of longer and larger-diameter screws for salvage might be clinically applicable in cases with completely absent pedicles. However, the pullout strength seems to be higher for longer screws than for larger-diameter screws (Fig 9), which implies that salvage strategies that use longer screws are more practical. The interaction between embedded bone volume and pedicle screw fixation strength has not been thoroughly studied. Previous studies have shown wide anatomical variation in pedicles and vertebral body size and morphology across the human lumbar spine [35]. As a result, this study was designed to investigate the correlation between embedded bone volume and pullout force in the standard homogenous Sawbone testing model. Our results support the hypothesis that a higher screw pullout force is obtained with a larger embedded bone volume (Fig 12). Longer screws (6.0 mm × 50 mm) provided greater increases in pullout strength than larger-diameter screws (6.5 mm × 45 mm), which is comparable with the results obtained with our biomechanical test.

## Conclusions

This study presents an evolution of the well-established screw pullout test using the broken pedicle Sawbone testing model. The pedicle plays an important role in contributing to screw pullout strength. However, once the pedicle is broken, a change to a longer or larger-diameter screw might be necessary. Our image quantification data of embedded bone volume were consistent with the biomechanical testing results, which indicated that relative pullout strength might be converted from the embedded bone volume observed in a standard homogeneous spinal bone surrogate.

## Supporting information

S1 Files The original data used to create the figures for this manuscript can be found in the supporting-information file. (PDF)
