# Supplementary material for: Biomechanical study of the fixation stability of broken pedicle screws and subsequent strategies
Source: PLoS One. 2019 Jun 28;14(6):e0219189. doi: 10.1371/journal.pone.0219189 (PMC6599116; doi:10.1371/journal.pone.0219189)
Supplement: S1 Files — (PDF) [file pone.0219189.s001.pdf]

Typical Force VS. Displacement Curve for all groups

IP - 6x45 mm		IP - 6x50mm		IP - 6.5x45 mm		SP - 6x45 mm		SP - 6x50 mm		SP - 6.5x45 mm		NP - 6x45 mm		NP - 6x50 mm		NP - 6.5x45 mm	
mm	N	mm	N	mm	N	mm	N	mm	N	mm	N	mm	N	mm	N	mm	N
0	23.56032	0	124.8543	0	289.5764	0	63.66838	0	48.97746	0	69.29893	0	14.95183	0	26.91601	0	58.35481
0.047614	96.45955	0.051172	167.4776	0.004994	428.0926	0.023049	85.52731	0.034226	75.31051	0.033849	106.7062	0.055455	40.5014	0.042006	88.61865	0.040902	92.87885
0.099003	195.1229	0.087071	241.6445	0.05928	526.236	0.064856	98.51116	0.049613	107.9513	0.068276	161.4736	0.118367	47.25706	0.082467	138.6467	0.076649	141.9337
0.148829	258.2606	0.130534	323.6169	0.117831	603.5016	0.113366	114.8919	0.070168	150.3875	0.105523	213.6399	0.175917	50.8995	0.128482	195.8358	0.117384	188.6134
0.198647	323.1053	0.184371	433.7964	0.168975	674.8085	0.153626	136.2032	0.093423	200.1155	0.148657	258.9948	0.230961	51.47497	0.184639	254.946	0.349907	403.1601
0.246902	382.9779	0.232981	558.2854	0.219431	742.7217	0.189585	178.8474	0.12181	242.5378	0.1978	296.1346	0.279763	58.43942	0.235543	310.8802	0.401696	396.3647
0.297068	441.7728	0.284238	674.3692	0.272585	800.7807	0.220185	232.3913	0.156151	287.9232	0.249897	333.7566	0.33073	84.81319	0.285277	362.2438	0.432129	408.2696
0.346664	495.6157	0.334456	770.3194	0.324576	854.7024	0.256915	288.4113	0.199903	342.4295	0.309241	373.5075	0.373625	114.7939	0.338495	397.081	0.466981	418.0424
0.396209	545.2935	0.385648	860.9507	0.37653	870.1013	0.300396	342.8759	0.245738	389.6414	0.421375	422.0046	0.418895	135.1906	0.38994	416.6749	0.505653	425.2351
0.445314	593.6762	0.437256	941.3077	0.429238	919.6799	0.356372	382.3555	0.29589	434.2447	0.490996	442.0539	0.470123	156.2962	0.444053	418.1596	0.542731	426.9874
0.497979	639.6333	0.489555	1002.619	0.47853	963.4693	0.414412	413.1792	0.350073	469.8798	0.797917	352.5425	0.523244	178.511	0.497568	417.8113	0.582669	423.826
0.545879	684.3714	0.53958	1045.246	0.529925	986.3426	0.465589	436.5966	0.41018	490.2868	0.806898	346.3787	0.575043	197.6225	0.54884	413.3832	0.624288	418.3594
0.596796	724.0568	0.590404	1083.56	0.583387	960.9591	0.520246	451.4914	0.478208	496.0622	0.830103	362.1348	0.626493	214.2922	0.600399	408.3894	0.66581	414.494
0.646365	764.6178	0.641584	1117.038	0.63919	427.8135	0.578631	457.8632	0.549158	493.8156	0.859272	370.3442	0.675012	229.3488	0.653527	401.8313	0.708553	408.657
0.695682	803.1458	0.691898	1146.323	0.73376	376.3073	0.641312	455.1335	0.623564	475.2547	0.892782	367.754	0.728171	240.4786	0.704227	394.2846	0.753451	399.9023
0.746233	846.5394	0.740732	1173.939	0.761974	369.9948	0.701943	451.7188	0.70897	434.4224	0.928567	356.7342	0.780567	246.7402	0.7562	388.9824	0.79673	388.0271
0.796267	882.1154	0.792651	1200.378	0.798008	358.9744	0.761542	445.5221	0.773472	412.2815	0.9683	346.0046	0.831138	254.5424	0.808802	378.3163	0.84159	374.5074
0.846221	918.4732	0.844056	1218.155	0.845327	336.984	0.83007	433.6619	0.834171	401.9121	1.002981	337.0259	0.881881	257.1115	0.85925	369.4173	0.8882	358.9588
0.897246	953.3923	0.894452	1232.658	0.897862	312.9535	0.8997	418.0948	0.892326	387.7828	1.039664	330.3746	0.933245	255.9809	0.908849	357.6334	0.933798	346.4308
0.944868	984.5633	0.944759	1241.667	0.951921	296.5175	0.954399	399.2517	0.952079	374.8922	1.077926	323.7475	0.985098	250.3631	0.96043	352.6641	0.979375	333.1748
0.996269	1015.937	0.99881	1242.341	1.005278	281.0533	1.013656	381.8632	1.015184	366.4502	1.117346	313.9665	1.036669	243.7781	1.009523	346.2667	1.026882	319.1857
1.048201	1044.658	1.051469	1227.852	1.057566	269.6033	1.068764	366.2763	1.097742	351.5018	1.159604	302.2879	1.088565	236.4967	1.060995	337.1895	1.073996	306.2359
1.09769	1068.271	1.127735	856.9204	1.108697	257.4222	1.118955	357.049	1.153452	335.9388	1.200973	292.6644	1.140021	229.093	1.110578	323.3158	1.122415	292.6004
1.148202	1089.47	1.166716	798.5698	1.158603	246.1466	1.173594	349.267	1.21194	322.2472	1.243436	285.7033	1.190713	221.6255	1.161231	310.4154	1.168775	282.7978
1.198972	1107.83	1.210345	791.3427	1.208817	232.8355	1.225425	338.369	1.263829	312.1731	1.287876	279.931	1.241469	215.5323	1.211724	303.2498	1.215793	272.4722
1.250488	1122.275	1.260574	772.0566	1.259773	223.156	1.277004	328.7405	1.313078	305.4358	1.33182	269.582	1.291002	202.2523	1.260055	288.9662	1.264479	264.3509
1.300685	1132.968	1.314215	733.4586	1.308326	213.5076	1.331804	318.3293	1.36407	295.5454	1.377439	261.7353	1.343194	195.1359	1.310487	277.1592	1.312733	255.028
1.349809	1143.849	1.370508	672.933	1.358947	201.6763	1.384277	304.848	1.418737	283.4755	1.422992	255.0809	1.395548	187.8864	1.361132	269.2999	1.361741	241.6669
1.415975	780.7443	1.419821	619.7045	1.408461	193.3868	1.437213	292.799	1.4722	274.0518	1.467414	247.6364	1.447081	181.6437	1.409309	262.382	1.412049	228.0213
1.466031	702.7363	1.466364	571.1603	1.45687	184.2742	1.486158	283.1497	1.520151	267.945	1.513928	236.0582	1.498349	175.3506	1.458395	258.3537	1.459167	218.1743
1.517426	539.9375	1.513946	549.6361	1.506639	175.5597	1.535857	275.2983	1.569861	263.4792	1.564895	227.4241	1.547236	168.764	1.50796	246.0104	1.508347	209.7953
1.565854	545.8202	1.559747	534.4408	1.55554	165.9082	1.58565	265.7768	1.620175	253.1203	1.609156	220.8244	1.598569	161.0502	1.558129	237.4848	1.55789	202.07
1.614583	542.5215	1.608675	510.652	1.603292	156.6029	1.634123	260.4317	1.673189	244.1189	1.656237	218.4308	1.648012	154.1084	1.607533	228.4063	1.607037	194.3606
1.666649	538.8132	1.663931	473.8702	1.652848	147.4234	1.683236	255.544	1.723054	235.767	1.702648	212.7206	1.69635	148.3899	1.657364	219.0881	1.656427	187.1179
1.71424	531.1698	1.709852	462.1338	1.703162	131.8145	1.732338	247.9499	1.77232	228.5342	1.75129	206.5123	1.74515	141.7253	1.709508	211.047	1.70537	178.9859
1.761808	531.1917	1.757223	454.5105	1.752592	122.1088	1.785605	238.8273	1.821296	223.2892	1.800138	202.2885	1.794549	135.6874	1.756217	204.9094	1.755571	174.5371
1.81275	526.7702	1.80436	450.8579	1.803158	117.5389	1.836209	229.7535	1.871275	218.0844	1.849164	198.5558	1.84478	130.5616	1.806611	196.8383	1.805386	171.117
1.862481	520.9202	1.855327	443.0739	1.853571	113.5472	1.884451	224.3043	1.919662	212.7415	1.896545	197.3286	1.893858	126.9814	1.855905	191.0229	1.85388	166.6191
1.92024	324.688	1.904918	429.8511	1.900389	109.6339	1.935165	217.0234	1.970574	204.7268	1.943448	194.8338	1.943818	125.4098	1.906612	184.8544	1.90368	162.0966
1.96637	315.1443	1.957543	405.3253	1.948924	107.2024	1.982989	211.4248	2.019817	198.706	1.992966	188.8487	1.991415	126.1934	1.953956	178.3683	1.953183	157.8206
2.015281	305.5323	2.011887	377.7774	1.998422	105.0986	2.032106	205.6387	2.067778	194.063	2.041662	182.8515	2.04023	124.7495	2.004426	170.9037	2.002448	152.9052
2.064965	288.3139	2.059478	364.069	2.047222	99.85807	2.078185	205.5192	2.114047	192.4392	2.092691	178.9208	2.088819	124.4289	2.055757	162.1712	2.051218	149.2419
2.115825	277.3344	2.105215	357.8328	2.097877	95.85065	2.122153	206.6458	2.161185	189.7347	2.140349	176.5199	2.138575	120.5924	2.105637	155.6819	2.101728	147.494
2.166838	256.8071	2.155656	351.7742	2.147978	89.69232	2.161483	210.0736	2.20964	186.3906	2.189514	172.4585	2.188887	119.4873	2.15494	149.6852	2.151506	145.2221
2.214385	238.3391	2.204595	344.1222	2.196295	86.46549	2.203187	213.187	2.257324	184.8824	2.238375	169.6314	2.238575	117.7905	2.204416	143.212	2.20034	143.511
2.263555	225.7693	2.255751	339.7355	2.247707	83.88688	2.250295	218.0965	2.30448	180.3324	2.288792	167.1338	2.292129	118.689	2.255056	138.8047	2.251592	139.1376
2.313019	213.0109	2.304129	334.821	2.297025	79.23835	2.300401	220.3908	2.354132	178.6462	2.338699	166.3018	2.343188	118.947	2.305595	133.9146	2.300682	144.0735
2.362812	205.1945	2.355612	328.4811	2.344039	77.35741	2.352827	219.101	2.407199	172.8424	2.38823	164.6057	2.39118	117.293	2.354338	126.0568	2.350154	142.3527
2.411403	195.9011	2.406154	324.135	2.394719	75.64485	2.410571	214.3057	2.460561	162.8399	2.436706	161.3538	2.43911	114.7876	2.405184	121.617	2.400181	135.2951
2.462617	187.8179	2.453332	323.5756	2.445856	69.06801	2.469501	208.9643	2.512159	154.8696	2.487114	155.723	2.490422	113.1993	2.452359	118.2057	2.450495	129.792
2.512737	179.8804	2.50125	323.1489	2.495697	68.41174	2.522461	202.0285	2.56333	147.1049	2.537093	155.4144	2.539868	111.7768	2.502224	116.6394	2.499864	126.8564
2.562113	172.4467	2.550546	321.9783	2.546276	65.82688	2.574044	195.9111	2.612698	141.9487	2.587335	151.7246	2.589639	108.0401	2.553002	113.8375	2.550157	122.7402
2.609993	169.1609	2.598453	318.7934	2.597645	62.88154	2.624837	188.5316	2.662122	137.3159	2.635847	146.7769	2.640091	103.5127	2.596474	110.7652	2.598065	120.3097

2.659863	163.8108	2.648668	315.5289	2.647024	61.15325	2.674594	182.5942	2.711854	133.0809	2.685687	140.7267	2.689727	99.17864	2.644586	108.2727	2.650565	115.9314
2.708071	158.1173	2.699702	314.0894	2.695355	56.33993	2.724092	179.1249	2.762103	127.8843	2.735406	138.5561	2.739963	94.35278	2.69262	106.2253	2.699161	112.5303
2.758878	153.4489	2.750791	308.9725	2.744733	52.12389	2.775507	176.5337	2.812424	120.6712	2.785282	132.7255	2.790078	89.35966	2.742628	104.4265	2.749169	109.8157
2.810117	146.6564	2.800801	303.4366	2.794628	49.87365	2.823834	169.6959	2.864043	112.5931	2.835546	131.2181	2.841206	82.13888	2.796996	102.6228	2.799651	105.241
2.859258	142.9081	2.851868	300.7177	2.8436	45.06828	2.873059	166.4901	2.913117	108.9338	2.885257	126.6679	2.890901	76.12449	2.852791	98.96099	2.849605	103.72
2.908801	138.1085	2.900682	300.4354	2.895313	43.99581	2.922595	160.6374	2.957724	107.701	2.936353	122.1981	2.939616	74.17823	2.905282	97.53415	2.898984	101.424
2.958002	143.2023	2.950251	304.8831	2.944337	40.78291	2.971751	158.9535	3.003651	106.2876	2.983537	118.6774	2.989749	71.41712	2.954408	95.78797	2.948971	97.18379
3.008296	155.0459	2.998973	303.5554	2.995205	39.49478	3.021631	154.8374	3.050786	104.2645	3.033726	116.6154	3.03875	68.60136	3.004995	95.85782	2.998203	95.91039
3.05694	154.5325	3.049099	303.8828	3.045014	37.79828	3.064746	153.2129	3.096901	104.4874	3.085381	113.1813	3.08939	66.09811	3.055463	94.93376	3.049099	92.78734
3.109301	156.5197	3.097661	299.9387	3.09517	36.56559	3.109697	150.6726	3.141614	105.1996	3.135185	111.2347	3.139919	61.46741	3.103486	94.49124	3.098496	92.15749
3.155263	159.4661	3.148138	294.2899	3.144831	35.60334	3.158952	152.5547	3.184516	105.9727	3.184059	106.978	3.19005	59.19363	3.150673	94.2674	3.147751	91.27702
3.206513	162.7038	3.200417	289.8507	3.194995	34.18811	3.207956	153.8022	3.229336	106.6774	3.234315	106.4728	3.240454	56.67885	3.200646	95.47277	3.198832	89.50266
3.255832	165.0716	3.251316	280.9905	3.246339	31.03362	3.259034	153.307	3.278168	104.8998	3.284713	103.1487	3.289087	55.14426	3.250808	94.85143	3.248425	88.6858
3.305534	166.7238	3.303073	276.4297	3.293299	31.25094	3.31129	151.913	3.32843	104.2172	3.333338	102.453	3.338497	50.90137	3.299005	95.99961	3.298529	87.68947
3.357038	168.6812	3.353839	270.1713	3.344935	29.92175	3.36372	150.1071	3.377535	105.6955	3.383875	99.09876	3.387443	50.71771	3.347281	95.35383	3.348276	87.21845
3.404769	172.5617	3.404405	267.6692	3.393972	31.2794	3.414186	148.4424	3.427139	106.2514	3.432468	98.67286	3.436777	48.32246	3.397699	96.49698	3.398537	85.12764
3.455766	174.645	3.453018	265.2679	3.443369	30.39156	3.461941	148.6808	3.474612	104.7191	3.482093	94.83466	3.485987	47.52053	3.449213	95.82014	3.447993	85.42933
3.505688	174.0989	3.502733	262.1617	3.493497	29.44842	3.512033	146.1055	3.521667	104.1219	3.534459	95.14636	3.536459	42.41783	3.499402	92.98879	3.498091	83.30262
3.555646	175.1847	3.552351	253.9928	3.543611	30.13754	3.562628	144.2336	3.572113	105.1664	3.582524	94.31458	3.586073	43.10552	3.548815	91.26216	3.547833	82.88678
3.605557	178.1053	3.603273	250.3629	3.593273	28.6994	3.612772	142.5682	3.62191	103.9807	3.631497	94.19044	3.633829	41.68199	3.599985	89.25824	3.598331	82.24189
3.655503	178.3403	3.654271	246.717	3.644989	31.28582	3.663569	140.2568	3.675187	102.1838	3.682753	93.80984	3.683293	42.60205	3.650817	87.00169	3.648437	81.23033
3.704397	177.1181	3.703558	246.6874	3.694296	30.51659	3.714452	134.7776	3.727477	101.2995	3.731637	94.50473	3.733291	40.08361	3.70154	86.07116	3.69852	79.96019
3.755338	175.3643	3.75349	246.5645	3.74516	32.0231	3.767308	132.4951	3.782682	97.22468	3.781611	92.76597	3.783269	39.75928	3.749786	83.69079	3.747886	79.80354
3.805529	168.5948	3.801216	250.7024	3.794521	30.81247	3.81618	127.4688	3.837406	96.40423	3.833395	92.09733	3.833273	38.69312	3.799646	82.31281	3.798156	77.21763
3.855591	162.0099	3.849864	249.9595	3.844797	31.55595	3.864612	127.96	3.889655	97.07157	3.882225	92.96769	3.882874	40.28154	3.851643	79.07809	3.84721	75.4837
3.904424	155.8131	3.899631	247.3568	3.893785	31.87177	3.913093	126.8058	3.937579	96.11543	3.933567	93.83209	3.933215	39.37017	3.901883	76.70973	3.897525	74.63894
3.95571	147.0057	3.949609	235.0019	3.944003	31.54915	3.958896	127.4056	3.982612	96.62532	3.983433	92.2167	3.982594	42.11073	3.951701	75.31033	3.94779	73.42044
4.00742	142.9281	4.000298	228.5116	3.992915	31.80508	4.010226	123.7562	4.028234	96.91148	4.032835	93.13712	4.033914	40.75414	4.003364	72.17252	3.997985	70.78126
4.056731	133.6145	4.054016	227.955	4.041988	29.84729	4.057534	127.0367	4.074816	97.20507	4.084557	89.742	4.083884	42.69101	4.052346	69.12844	4.048159	69.37157
4.106749	127.08	4.103243	223.9765	4.091699	28.8424	4.10386	129.2896	4.121314	95.77703	4.131751	91.28726	4.135211	41.7629	4.104533	68.46404	4.097586	67.41439
4.155647	119.9519	4.15258	220.6779	4.143492	29.51118	4.145158	131.4571	4.171819	96.36423	4.182306	89.29492	4.185994	43.33526	4.153767	67.43735	4.147302	68.84365
4.20605	113.4293	4.202858	214.1075	4.191847	28.45846	4.189197	133.1219	4.225594	93.86372	4.231399	90.43197	4.237186	42.03739	4.202767	67.55823	4.196903	69.78028
4.255114	108.5456	4.251872	208.7778	4.241917	27.9452	4.239488	135.3344	4.281845	92.02068	4.281767	89.82333	4.286739	43.58389	4.252694	64.76659	4.248142	70.1211
4.306647	100.933	4.301745	205.3882	4.294033	26.71671	4.293086	133.8356	4.340783	89.66322	4.332031	89.44318	4.336106	43.07756	4.302142	63.27681	4.297313	68.15451
4.355861	99.06352	4.351531	203.2373	4.344027	26.63232	4.349604	133.006	4.392057	87.8436	4.382323	89.10146	4.384572	44.16357	4.351685	62.77631	4.347268	67.96513
4.40574	93.65682	4.401661	195.7261	4.393473	27.15754	4.406666	131.425	4.437736	85.13839	4.432301	89.56074	4.434334	42.96938	4.401123	62.59135	4.397728	67.26356
4.456065	89.93126	4.451128	193.1815	4.442645	25.27809	4.459789	127.7312	4.486022	83.57563	4.482463	87.24153	4.485028	41.79254	4.449745	61.46585	4.447208	66.83711
4.506333	87.85352	4.500944	188.9847	4.491646	26.04546	4.512781	125.7256	4.534009	82.82229	4.533514	87.09468	4.53779	39.43206	4.498854	60.08721	4.497845	66.42188
4.5562	83.83943	4.551197	186.8539	4.541128	23.09917	4.562011	126.089	4.581363	83.99329	4.582514	86.80676	4.585033	40.35048	4.547835	60.859	4.548093	65.85354
4.605614	82.53902	4.601752	180.8797	4.592603	22.67872	4.611873	125.0388	4.630696	85.8835	4.632403	85.0193	4.635861	39.616	4.596982	60.67739	4.597167	64.67816
4.655758	76.70555	4.651572	177.6646	4.642093	22.07439	4.659663	124.2725	4.681325	79.40959	4.682109	84.95008	4.685495	40.51679	4.6476	60.58049	4.64741	65.7558
4.706697	72.17913	4.701361	177.5444	4.691966	22.08593	4.710328	122.6828	4.736488	74.79177	4.733218	83.11735	4.737023	36.82901	4.697903	60.24749	4.697513	65.62294
4.756206	63.09594	4.751185	175.9805	4.742408	20.46981	4.759046	123.281	4.788822	72.79711	4.782066	80.86503	4.78676	36.15725	4.748931	60.30202	4.747919	67.00423
4.805096	58.35071	4.79912	176.1603	4.79298	19.60835	4.811465	120.7809	4.835016	72.72903	4.831955	80.26977	4.837669	34.11714	4.799616	59.74639	4.797029	63.56458
4.855454	59.11686	4.845798	174.1781	4.843171	19.06937	4.86103	120.0576	4.882907	71.73353	4.882532	79.24881	4.888379	35.48772	4.848728	59.13582	4.848206	62.27992
4.905067	64.55109	4.897365	175.1909	4.892716	19.07381	4.908851	118.6443	4.929781	72.09219	4.932435	79.47573	4.936799	33.81288	4.898753	59.29591	4.896904	59.99576
4.954067	74.19018	4.948188	173.0131	4.94336	18.2243	4.958821	118.8129	4.979266	68.90772	4.982534	78.06346	4.989198	32.63713	4.948434	59.10955	4.94705	59.53345
5.004386	76.23159	4.99772	172.1371	4.992761	18.35525	5.007584	119.5	5.030264	69.34363	5.03161	76.80223	5.038662	30.35041	4.998502	61.55358	4.99759	58.95584
5.054147	77.11817	5.049319	166.7679	5.042458	16.92477	5.056112	119.3438	5.078092	68.57896	5.082312	77.1507	5.08949	31.39698	5.047758	60.85876	5.046511	57.37334
5.104349	79.74226	5.100987	164.5266	5.091617	16.95532	5.108191	118.557	5.129589	68.84546	5.129291	76.56652	5.139102	28.04194	5.097983	60.47697	5.096412	56.71098
5.154414	83.68292	5.152933	163.8874	5.142871	15.8402	5.156346	119.3545	5.179453	66.44651	5.177253	75.73022	5.187722	27.68398	5.150347	60.10332	5.146537	56.68718
5.204651	87.2457	5.200832	161.4738	5.19198	17.34528	5.204324	119.5917	5.228693	65.89033	5.226713	76.15703	5.239192	25.38301	5.198398	59.91627	5.196947	55.57118
5.254694	91.20041	5.250458	159.2188	5.242679	16.12669	5.255844	120.0527	5.27664	65.07435	5.276401	73.04014	5.288826	26.2782	5.249345	58.90913	5.247225	54.84475
5.304398	95.76506	5.298617	158.5343	5.290805	15.54608	5.306167	118.0108	5.323122	67.31981	5.326205	73.05733	5.338824	23.94789	5.301534	59.36823	5.297455	55.0177
5.354995	99.81818	5.349382	158.8796	5.343424	16.0858	5.355283	117.7174	5.367605	65.34252	5.375424	71.52311	5.386462	25.72252	5.350804	58.13423	5.346714	55.05592
5.404331	102.7842	5.399716	160.1591	5.394692	14.43922	5.406028	116.7247	5.417085	64.49655	5.426896	70.81385	5.437505	22.68925	5.398902	56.68299	5.397626	54.59162
5.454014	105.9165	5.448125	160.0492	5.444568	15.06805	5.454109	118.2736	5.466932	64.20584	5.47809	68.69774	5.486332	22.9496	5.451684	56.70204	5.44681	54.83781

5.503903	109.0406	5.498991	157.8108	5.494687	15.56012	5.502708	116.7188	5.514741	63.67178	5.528846	67.44467	5.535657	21.57018	5.501139	56.64667	5.496791	52.53353
5.553355	115.6166	5.549222	158.8652	5.543344	16.29096	5.555789	115.3706	5.563482	64.27421	5.579701	67.05163	5.585646	21.87663	5.55099	55.94134	5.54656	51.71169
5.604098	117.2127	5.598074	160.1274	5.594463	15.01185	5.610021	110.9567	5.615072	62.55512	5.629264	67.00668	5.635334	19.853	5.602925	53.85648	5.59733	51.73641
5.653916	119.2883	5.649075	162.9233	5.644591	18.06822	5.661871	106.7257	5.669844	61.71544	5.679546	64.1275	5.685004	19.53719	5.651184	56.77152	5.647113	50.50604
5.703636	121.4341	5.698834	164.4263	5.693185	17.02639	5.713016	103.0353	5.721978	58.86094	5.730276	63.69863	5.736429	17.6758	5.702479	54.71487	5.696415	51.33939
5.753502	120.6212	5.747451	167.2938	5.743654	18.68111	5.759306	100.0223	5.775721	61.36135	5.782579	61.33658	5.786826	17.6732	5.75256	53.05754	5.74727	50.52816
5.803492	118.6594	5.797394	168.8982	5.793903	17.24705	5.8069	100.905	5.826432	60.80647	5.831596	61.72465	5.836739	18.99949	5.803632	51.05096	5.795712	48.56917
5.853792	115.6845	5.84745	170.2519	5.841065	16.86084	5.856668	98.29108	5.876652	61.39008	5.882017	61.43872	5.88815	18.26985	5.85313	51.82759	5.84641	50.38763
5.902544	113.3757	5.897168	171.8407	5.889905	15.79019	5.909407	98.2096	5.926549	59.89396	5.931195	62.81424	5.938427	19.24536	5.904932	49.89682	5.895809	49.832
5.954255	105.0263	5.946717	171.5029	5.940796	16.05707	5.956806	99.14548	5.974788	60.55292	5.982296	60.13941	5.987478	19.25882	5.952678	47.53615	5.946078	49.25556
6.004092	100.0207	5.996742	169.2872	5.990888	16.9341	6.005481	99.51689	6.022745	59.95087	6.031631	60.63463	6.037664	20.24784	6.002142	44.99969	5.997036	47.83817
6.053998	91.63939	6.046823	167.9493	6.040698	16.54297	6.051817	101.1292	6.069434	62.39489	6.08072	60.40346	6.086876	20.21398	6.051794	45.08752	6.046276	48.7017
6.104751	87.4748	6.099886	169.3871	6.091144	15.22997	6.093487	103.1312	6.115478	61.46617	6.130949	61.33153	6.136892	19.90697	6.102381	43.72988	6.096241	50.94484
6.154261	82.14335	6.149331	168.9288	6.140989	14.11307	6.136411	106.6064	6.1642	59.45735	6.179729	59.44896	6.185648	22.36944	6.146546	43.24634	6.145272	49.8604
6.205194	79.29527	6.199942	167.047	6.191795	14.98386	6.182525	107.8571	6.216178	58.22475	6.229556	60.19808	6.234438	20.5277	6.196124	44.39605	6.195038	49.60778
6.254084	75.66251	6.249495	162.8469	6.241762	14.65604	6.237424	106.9889	6.270724	58.77717	6.278904	59.88925	6.28554	20.18199	6.249889	41.80903	6.245566	47.43636
6.304748	71.54578	6.300057	159.8252	6.291351	14.02911	6.292704	104.3997	6.324419	56.31898	6.32953	60.67013	6.334596	21.09492	6.301021	42.33699	6.295919	47.9354
6.354757	70.15378	6.350202	153.9386	6.342148	13.53565	6.348717	103.5652	6.378719	54.9125	6.378973	60.31082	6.383449	20.10413	6.351876	41.60721	6.345745	48.38929
6.404178	67.16303	6.401114	149.7794	6.392639	12.03579	6.401351	102.6547	6.433853	52.84771	6.428231	60.54199	6.433618	19.31666	6.400917	41.42392	6.396712	47.36288
6.453009	66.76633	6.451077	145.0784	6.441466	12.70986	6.458393	101.4323	6.488654	51.89837	6.479734	58.13631	6.483584	17.87897	6.452684	39.4823	6.445631	46.11453
6.504098	63.466	6.500964	142.5461	6.49338	11.73797	6.510077	98.80769	6.536368	51.02512	6.529207	59.77517	6.532936	17.29308	6.500993	39.92273	6.496242	44.95508
6.553734	58.87435	6.550044	140.7284	6.541713	11.0364	6.561667	97.20973	6.580382	50.2706	6.580389	58.63025	6.584356	16.28501	6.54974	42.66962	6.544942	46.59472
6.602449	55.66664	6.601638	139.1861	6.593358	8.18577	6.610675	96.75115	6.629267	50.6009	6.630879	56.58187	6.633165	16.8792	6.601263	42.408	6.594396	45.6146
6.6536	51.78591	6.649199	136.1865	6.642714	9.79844	6.658694	97.98817	6.679384	48.72806	6.68062	54.49826	6.683863	13.7494	6.650629	41.72372	6.645835	46.62201
6.703049	46.81782	6.69884	135.8859	6.692483	9.17807	6.705216	97.99018	6.7294	48.57015	6.729642	55.50482	6.735198	14.21473	6.699646	42.65406	6.694212	45.9421
6.754292	43.25882	6.746578	135.8338	6.742315	9.03931	6.75184	96.47179	6.7811	46.89666	6.780736	54.60246	6.784186	11.3491	6.750231	41.96698	6.74593	44.14143
6.804546	43.13728	6.796331	136.3824	6.790769	8.47804	6.801561	95.82853	6.832506	45.45283	6.829825	55.0319	6.835301	13.05188	6.800339	42.22733	6.795	44.04173
6.852952	47.06192	6.845619	135.3515	6.840543	6.69905	6.852105	98.35503	6.882183	43.22571	6.879571	52.45415	6.884716	12.11097	6.849696	42.0786	6.844949	43.17486
6.905491	54.8807	6.897068	135.2747	6.891389	5.74124	6.900986	96.56097	6.931906	43.83801	6.929492	52.07818	6.933992	12.55018	6.899652	40.96516	6.894467	42.64684
6.953067	57.21594	6.946424	134.2299	6.941091	6.88806	6.952498	95.56487	6.979259	42.29527	6.97956	50.78984	6.984739	12.47204	6.950126	42.14225	6.944709	42.10961
7.003737	58.45209	6.998384	135.6572	6.991147	6.01914	7.002112	95.46727	7.029037	42.85518	7.029996	51.019	7.034113	10.84129	6.998267	42.64265	6.995192	42.32866
7.053859	58.43337	7.048032	135.2026	7.041104	5.42179	7.049632	95.95537	7.077991	40.98616	7.079255	50.21114	7.082576	10.88129	7.049342	40.98728	7.044118	40.96874
7.104097	60.51213	7.096819	135.4615	7.092632	5.11724	7.096604	98.09458	7.129222	39.03001	7.129287	50.32265	7.13038	9.977259	7.098589	40.81093	7.095402	40.91407
7.154387	64.52706	7.148036	135.2785	7.141096	5.23025	7.14224	98.43102	7.178817	37.03856	7.179317	47.20906	7.181616	11.16078	7.148838	39.63378	7.145114	38.29952
7.20199	67.2881	7.19665	135.1436	7.190452	5.36376	7.188841	98.52578	7.227508	38.38847	7.229942	48.64523	7.231574	8.969288	7.198709	40.39733	7.195728	39.59741
7.252634	69.7277	7.247829	135.7014	7.240912	5.92032	7.241014	98.94399	7.277946	37.50506	7.278351	48.33917	7.281136	9.360723	7.249322	41.00618	7.244001	39.61422
7.304564	71.03514	7.297466	137.3052	7.291884	5.67585	7.292614	98.29617	7.325444	38.03382	7.328881	47.26302	7.33257	8.652546	7.299435	41.0534	7.294822	39.78284
7.353571	74.07856	7.346861	136.8823	7.341832	5.13539	7.341045	98.95228	7.372461	38.33175	7.379602	46.40906	7.382815	8.605048	7.350684	39.68589	7.344783	36.93458
7.403631	78.16297	7.397222	136.7559	7.391808	5.41535	7.393686	99.03564	7.419742	39.96049	7.429562	47.15051	7.431355	9.578373	7.398608	39.08328	7.394352	36.46696
7.451945	79.67334	7.44672	133.8837	7.440352	6.44401	7.445309	96.75296	7.46578	40.57558	7.480658	47.85659	7.481635	8.634757	7.448647	40.20305	7.445687	36.85355
7.50397	82.06782	7.496472	135.1025	7.490152	7.26902	7.49809	95.63323	7.514995	40.68082	7.530631	47.62971	7.532108	7.212861	7.497243	41.0039	7.494898	38.1188
7.55171	86.3126	7.546472	135.4827	7.542532	4.94927	7.554384	93.74446	7.563912	38.85347	7.579299	47.7552	7.581249	6.802985	7.546296	38.4164	7.544166	38.98209
7.602819	90.82127	7.595718	135.3863	7.591902	6.12834	7.609709	91.20973	7.615618	38.37854	7.629765	46.71316	7.632486	7.182546	7.597804	38.55252	7.595428	38.0537
7.651849	92.57244	7.644879	137.0103	7.641981	5.14147	7.668932	85.47008	7.666186	39.07704	7.680259	48.275	7.683404	6.571924	7.649707	36.81444	7.645226	37.49663
7.70155	93.06385	7.693841	136.8026	7.692235	7.92062	7.71974	85.18919	7.71782	36.61127	7.730529	48.32675	7.733316	5.630171	7.700667	37.18697	7.695778	38.80868
7.751698	93.06125	7.745589	137.8473	7.743691	6.69485	7.768122	84.31719	7.767997	39.55737	7.780576	48.43234	7.784324	4.724087	7.750158	35.76647	7.744532	37.91321
7.80186	94.56747	7.795248	136.3237	7.791239	6.16413	7.81411	84.23817	7.816381	36.70799	7.830681	48.01861	7.8346	7.492397	7.799019	34.67328	7.795563	37.15279
7.852542	92.13053	7.844382	135.9715	7.84265	5.04661	7.864284	83.4947	7.865589	36.65788	7.880511	48.24843	7.883997	8.076103	7.848874	34.61326	7.84489	36.75003
7.901759	89.62829	7.896079	133.5173	7.89237	6.40413	7.90991	84.5416	7.916329	36.0589	7.929336	48.46236	7.934484	7.842947	7.897648	33.72603	7.894292	37.2746
7.952295	84.09172	7.944031	135.3769	7.942893	5.53805	7.953885	84.81159	7.964176	36.96214	7.980019	48.57178	7.983921	7.572398	7.950175	33.6552	7.943794	37.33333
8.002218	77.06634	7.995202	133.6474	7.991998	6.74152	8.000064	87.95276	8.014558	35.92265	8.029208	48.27633	8.032887	6.220304	7.999123	33.74713	7.994713	37.81594
8.052501	70.80459	8.045735	135.1766	8.039707	5.26285	8.04369	89.00496	8.061611	34.57367	8.080084	47.93241	8.082642	7.530209	8.050109	32.36877	8.044496	38.03661
8.102737	68.3289	8.093857	130.8263	8.089678	6.15972	8.086272	91.93346	8.111213	35.20073	8.128635	47.00131	8.132945	8.442439	8.098347	30.77942	8.094596	38.04113
8.152636	63.34791	8.14469	129.8148	8.139914	5.26801	8.134264	93.71582	8.160991	36.20894	8.178097	47.92773	8.181933	7.961225	8.147823	30.80806	8.143425	37.78581
8.203438	61.66916	8.19612	127.0988	8.189467	5.73397	8.187322	94.7454	8.212242	34.64357	8.229293	47.06251	8.232707	8.193636	8.19677	31.22208	8.195431	37.67526
8.253093	59.42579	8.247035	126.2157	8.240417	4.71336	8.24072	94.43471	8.264143	35.67752	8.279865	48.45322	8.281858	7.111905	8.246237	30.33383	8.243685	37.92821
8.302429	56.84597	8.29761	124.6962	8.291195	5.1659	8.296189	93.0544	8.316819	32.58265	8.328735	48.01397	8.331855	8.392753	8.294365	30.73094	8.295366	38.66861

8.353303	54.45574	8.345358	122.6766	8.340574	5.42638	8.350494	92.06869	8.37085	31.36452	8.379241	47.65224	8.381727	7.763272	8.345805	30.5614	8.344727	37.51414
8.404047	53.21633	8.395587	121.5693	8.390635	6.22114	8.40514	92.21048	8.426603	32.28425	8.429001	47.07464	8.429684	8.15047	8.394217	30.37509	8.395159	37.4217
8.452641	50.17817	8.445498	118.8272	8.44123	5.27263	8.458178	90.92554	8.481658	29.63836	8.479002	48.1942	8.479591	6.689411	8.446328	30.71199	8.444112	38.44779
8.502942	46.17665	8.496894	118.8933	8.492255	5.07757	8.507183	90.19212	8.536578	29.7877	8.528957	46.96944	8.529159	6.318046	8.496811	30.24675	8.494809	39.19667
8.551929	43.13248	8.545583	114.7179	8.54114	4.29353	8.559021	88.49446	8.585755	26.97157	8.579206	46.06972	8.579494	5.137781	8.54759	28.5062	8.544419	39.64673
8.603349	37.89244	8.596522	113.7585	8.591964	4.95624	8.608867	87.89529	8.633191	27.32542	8.628998	46.16928	8.628603	6.060442	8.596413	29.43841	8.59395	39.10726
8.651509	34.71123	8.645911	113.7985	8.641926	3.84238	8.658305	88.2552	8.681663	28.45163	8.681173	44.83365	8.680037	5.125115	8.646025	31.2848	8.643802	38.38744

Maximal Pullout Strength (IP Group)

Pullout - (IP)	6 X 45 mm
	Max Pullout Force (N)
1	648.41
2	1105.1
3	1143.85
4	1145.3
5	1231.78

Pullout - (IP)	6 X 50 mm
	Max Pullout Force (N)
1	821.52
2	829.24
3	1026.7
4	1242.34
5	1436.47

Pullout - (IP)	6.5 X 45 mm
	Max Pullout Force (N)
1	840.65
2	891.4
3	958.76
4	1065.27
5	1341.56

Maximal Pullout Strength (NP Group)

Pullout - (NP)	6 X 45 mm
	Max Pullout Force (N)
1	257.11
2	301.56
3	307.94
4	334.88
5	372.97

Pullout - (NP)	6 X 50 mm
	Max Pullout Force (N)
1	368.24
2	414.85
3	418.16
4	432.09
5	490.49

Pullout - (NP)	6.5 X 45 mm
	Max Pullout Force (N)
1	315.62
2	426.32
3	426.98
4	409.91
5	449.33

Maximal Pullout Strength (SP Group)

Pullout - (SP)	6 X 45 mm
	Max Pullout Force (N)
1	369.74
2	393.25
3	443.12
4	457.86
5	517.07

Pullout - (SP)	6 X 50 mm
	Max Pullout Force (N)
1	435.01
2	446.61
3	496.06
4	517.27
5	563.94

Pullout - (SP)	6.5 X 45 mm
	Max Pullout Force (N)
1	357.89
2	417.98
3	424.43
4	442.05
5	647.58
